# MYB97, MYB101 and MYB120 Function as Male Factors That Control Pollen Tube-Synergid Interaction in *Arabidopsis thaliana* Fertilization

**DOI:** 10.1371/journal.pgen.1003933

**Published:** 2013-11-21

**Authors:** Yan Liang, Ze-Min Tan, Lei Zhu, Qian-Kun Niu, Jing-Jing Zhou, Meng Li, Li-Qun Chen, Xue-Qin Zhang, De Ye

**Affiliations:** 1 State Key Laboratory of Plant Physiology and Biochemistry, College of Biological Sciences, China Agricultural University, Beijing, China; 2 National Center for Plant Gene Research (Beijing), Institute of Genetics and Developmental Biology, Beijing, China; Nagoya University, Japan

## Abstract

Pollen tube reception involves a pollen tube-synergid interaction that controls the discharge of sperm cells into the embryo sac during plant fertilization. Despite its importance in the sexual reproduction of plants, little is known about the role of gene regulation in this process. We report here that the pollen-expressed transcription factors MYB97, MYB101 and MYB120 probably control genes whose encoded proteins play important roles in *Arabidopsis thaliana* pollen tube reception. They share a high amino acid sequence identity and are expressed mainly in mature pollen grains and pollen tubes. None of the single or double mutants of these three genes exhibited any visible defective phenotype. Although the *myb97 myb101 myb120* triple mutant was not defective in pollen development, pollen germination, pollen tube growth or tube guidance, the pollen tubes of the triple mutants exhibited uncontrolled growth and failed to discharge their sperm cells after entering the embryo sac. In addition, the *myb97 myb101 myb120* triple mutation significantly affected the expression of a group of pollen-expressed genes in mature pollen grains. All these results indicate that MYB97, MYB101 and MYB120 participate in pollen tube reception, possibly by controlling the expression of downstream genes.

## Author Summary

Pollen tube reception is an important step of fertilization and is controlled by interactions between the pollen tube and synergid. Components of both the pollen tube and synergid are believed to be involved in the process. Several proteins associated with this process have been identified in synergid cells. However, very little is known about the components contributed by the pollen tube. This work identified a group of *Arabidopsis* pollen-expressed MYB transcription factors, among which at least three members are involved in pollen tube reception. The *myb97 myb101 myb120* triple mutation caused overgrowth of the pollen tube into the embryo sac and disrupted sperm cell discharge, leading to failed fertilization. This study provides novel evidence demonstrating that male factors are involved in pollen tube reception.

## Introduction

In flowering plants, the proper development of male gametophytes is essential for successful fertilization during reproduction [1], [2]. The male gametophyte life cycle can be divided into two distinct phases: (1) a developmental phase, also known as the early developmental phase, which takes place in anther locules and leads to the formation of mature pollen grains; and (2) a functional or progamic phase, also called the later developmental phase, which begins when the pollen grains contact the stigma surface, continues with pollen tube growth and ends at double fertilization [1]. To date, many transcription factors involved in the early development of male gametophytes have been identified in efforts to understand the gene regulatory network involved in this process [1], [2]. However, the gene regulatory network that controls later development remains poorly understood.

The later developmental phase is delimited by pollination and fertilization. This process involves pollen hydration, germination, tube growth through the transmitting tissue, tube guidance, sperm cell discharge into the embryo sac and finally the fusion of male and female gametes. To date, four groups of transcription factors involved in this process have been identified. The AtMIKC* subgroup is composed of six members that belong to the MADS-box transcription factor family. Five of the six members in this subgroup (AGL30, AGL65, AGL66, AGL94 and AGL104) are expressed mainly in pollen and are expected to regulate the transcription associated with the later development of male gametophytes [3]–[6]. The AtMIKC* proteins interact with each other, forming five heterodimers that bind to the DNA motifs of their targets, namely AGL66-AGL30, AGL66-AGL65, AGL66-AGL94, AGL30-AGL104 and AGL104-AGL65. In the *agl66-1 agl104-2* double mutant, in which all the five heterodimers are absent, pollen viability was decreased. *In vivo*, pollen germination and pollen tube growth are also affected [7]. Microarray analyses demonstrated that this double mutation has a significant impact on pollen gene expression, indicating that the AtMIKC* proteins may function as upstream transcription factors during the later development of male gametophytes [7]. Another transcription factor found to be involved in the later development of male gametophytes is AtbZIP34, whose expression is detected in both male and female gametophytes during flower development [8]. A mutation in *AtbZIP34* caused defects in the exine, resulting in a lower pollen germination rate and slower pollen tube growth *in vitro* and *in vivo* [8]. The transcription factor AtWRKY34 is specifically expressed in pollen. Under cold treatment conditions, the *wrky34* pollen exhibited increased viability, germination efficiency and pollen tube growth rate *in vivo* relative to wild type pollen, indicating that *WRKY34* might function as a negative regulator of the cold response in mature pollen. Further analyses suggested that *WRKY34* acts downstream of the AtMIKC* genes in the cold stress response program [9]. In addition, the study shows that several MYB transcription factors are expressed in pollen tubes. Mutation in one (*MYB120*, *At5g55020*) of these genes caused defective pollen germination and tube growth *in vitro*. These data indicate that the pollen-expressed MYB transcription factors are also involved in the later development of male gametophyte [10].

Pollen tube reception is the final stage of male gametophyte development, which involves the bursting of the pollen tube to release sperm cells and the death of a synergid cell [11], [12]. However, our knowledge of the molecular and genetic mechanisms of pollen tube reception remains very limited. The first studies of these mechanisms focused on two female gametophytic mutants, *feronia* (*fer*) and *sirène* (*srn*). The pollen tubes of both *fer* and *srn* overgrow in the embryo sac and are unable to rupture (overgrowth phenotype) [13], [14]. Later, *fer* and *srn* were found to be alleles of the same gene, *FER* [15]. *FER* encodes a receptor-like protein kinase that localizes to the filiform apparatus of synergid cells. It has been proposed that the undefined signal produced from the pollen tube activates the FER receptor and then triggers a signal transduction cascade in the synergid that feeds back to the pollen tube and cause pollen tube rupture [11], [12], [15]. Three additional female gametophytic mutants, *lorelei* (*lre*), *scylla* (*syl*) and *nortia* (*nta*), were also found to exhibit *fer*-like phenotypes. The *LRE* gene encodes a putative glycosylphosphatidylinositol-anchored protein [16], [17]. The gene responsible for the *syl* phenotype has not been identified [18]. *NTA* encodes a Mildew Resistance Locus O family protein [19]. Recent studies showed that the FER pathway is important for the re-localization of NTA in the synergid cell upon the arrival of the pollen tube [19], [20]. Apart from the four female gametophytic mutants, a mutation in *ABSTINENCE BY MUTUAL CONSENT* (*AMC*) also causes a defect in pollen tube reception, but this defect only occurs when both the male and female gametophytes carry the mutant *amc* allele [21]. Maize (*Zea mays*) has also emerged as a model system for studying pollen tube reception in grasses. Zea mays embryo sac 4 (ZmES4), a defensin-like protein, is exclusively expressed in female gametophytes. Knocking down the expression of ZmES4 by RNA-interference resulted in the *fer*-like phenotype [22].

However, the male components controlling pollen tube reception are still poorly understood, although they are expected to exist, and only a few candidate genes have been identified to date. ANXUR1 (ANX1) and ANXUR2 (ANX2) are close homologues of FER, exhibit high-sequence homology to one another and are expressed in pollen. The *anx1 anx2* double-mutant pollen grains are able to germinate, but the pollen tubes rupture *in vitro* and in the transmitting tract *in vivo* and fail to reach the female gametophytes, indicating that *ANX1/ANX2* might function to ensure the proper timing of sperm discharge [23], [24]. That is, *ANX1/ANX2* maintain pollen tube growth in the transmitting tract until the tube reaches the female gametophyte; then, they are deactivated by an undefined signal from the synergid cell, which triggers the rupture of the pollen tubes [23]. In addition, *Autoinhibited Ca^2+^ ATPase 9* (*ACA9*) encodes a Ca^2+^ pump in *Arabidopsis* and is expressed in pollen tubes. The *aca9* mutant pollen tubes extend to the synergid cell and stop growing, but they do not rupture and discharge their sperm cells *in vivo* [25]. This finding indicates that the ion gradients in pollen tubes also play an essential role in sperm discharge. K+ channel Zea mays 1 (KZM1), a pollen tube-expressed shaker K^+^ channel, was discovered as a direct target of ZmES4 in maize. The interaction between ZmES4 and KZM1 triggers channel opening and a subsequent K^+^ influx that leads to pollen tube bursting [22]. Nevertheless, it remains unclear how these genes coordinate with each other to control pollen tube reception.

Here, we report the characterization of the novel male components MYB97, MYB101 and MYB120 that are involved in pollen tube reception. These proteins share a high amino acid sequence identity and are mainly expressed in mature pollen grains and pollen tubes. The *myb97 myb101 myb120* triple mutation causes the overgrowth of the mutant pollen tubes in embryo sac and disrupts the discharge of sperm cells into the embryo sac, leading to a significant reduction in fertility. Microarray analysis showed that the *myb97 myb101 myb120* triple mutation significantly affected the expression of a group of pollen-expressed genes in mature pollen grains. All these results indicate that the MYB transcription factors play important roles in pollen tube reception, possibly by controlling the expression of downstream genes.

## Results

### Identification and pollen-expression verification of the pollen-expressed *MYB* genes

**Figure 1:**
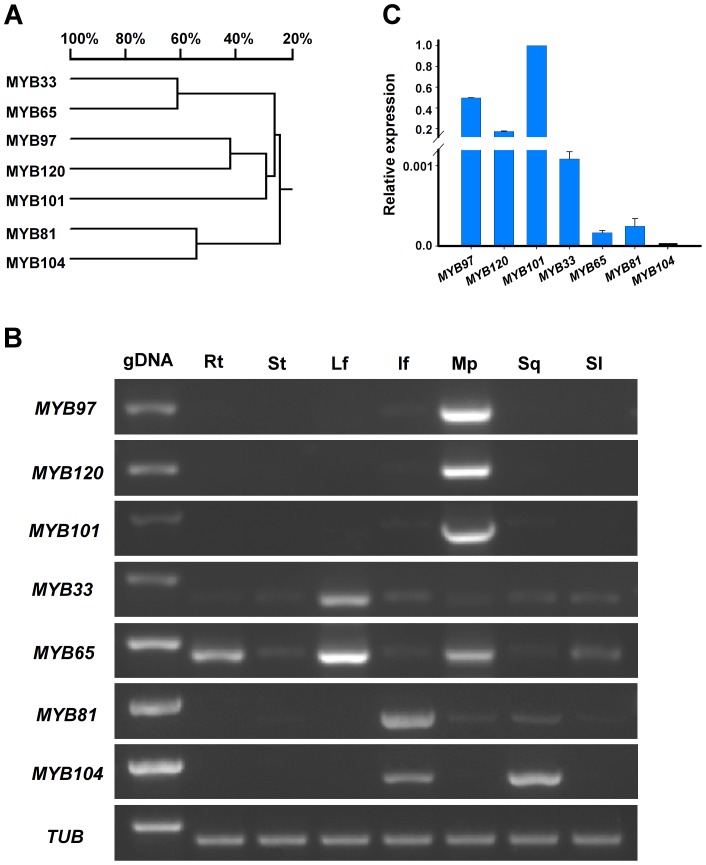
Identification of the *MYB* genes. (**A**) Phylogenetic analysis of the MYB proteins. (**B**) Expression patterns of the *MYB* genes, as revealed by RT-PCR. (**C**) Expression of the *MYB* genes in mature pollen grains, as revealed by qRT-PCR. gDNA, genomic DNA; Rt, roots; St, stems; Lf, leaves; If, inflorescences; Mp, mature pollen grains; Sq, siliques and Sl, seedlings.

MYB transcription factors play regulatory roles in diverse developmental processes in Arabidopsis. Several *MYB* genes have been identified as involved in the early development of male gametophytes and filament development (Table S1 and Text S1). Therefore, we speculate that the *MYB* genes might also function in the later development of male gametophytes. To study the gene regulation network that controls pollen tube growth, we manually searched the Arabidopsis gene expression profile data available in the TAIR databases (http://www.arabidopsis.org) for pollen-expressed transcription factor (PTF) genes [26], [27]. Seven MYB transcription factors were identified as expressed in mature pollen and pollen tubes, namely MYB33, MYB65, MYB81, MYB97, MYB101, MYB104 and MYB120 (Figure 1A and also see Table S2), which belong to the same subclass of the R2R3-MYB family [28]. Their expression patterns were first verified by RT-PCR of RNAs isolated from different tissues of wild type plants, including roots, stems, leaves, inflorescences, mature pollen grains, siliques and seedlings. The *MYB97*, *MYB101* and *MYB120* transcripts were detected mostly in mature pollen grains, weakly detected in inflorescences and siliques and not detected in the other tissues, suggesting that these three genes were expressed mainly in mature pollen grains. *MYB33* and *MYB65* were expressed in all the tissues tested, with significantly different levels in different tissues, including mature pollen grains. In particular, *MYB33* was expressed mostly in leaves, whereas *MYB65* was detected mainly in roots, leaves and pollen grains. The expression of *MYB81* was detected mainly in inflorescences and only weakly in mature pollen grains and siliques. *MYB104* was expressed mainly in the inflorescences and siliques (Figure 1B). Based on these results, the seven MYB protein*s* were classified into three subgroups: MYB97, MYB101 and MYB120 comprised the first subgroup, MYB33 and MYB65 comprised the second, and MYB81 and MYB104 comprised the third. Further quantitative reverse transcription-PCR (qRT-PCR) assays also confirmed that *MYB97*, *MYB101* and *MYB120* were expressed more highly in mature pollen, whereas the other four *MYB* genes were weakly expressed in pollen (Figure 1C). Therefore, the three genes in the first subgroup were considered the strongest candidate MYB members to have possible roles in pollen development and tube growth and were selected for further functional characterization.

**Figure 2:**
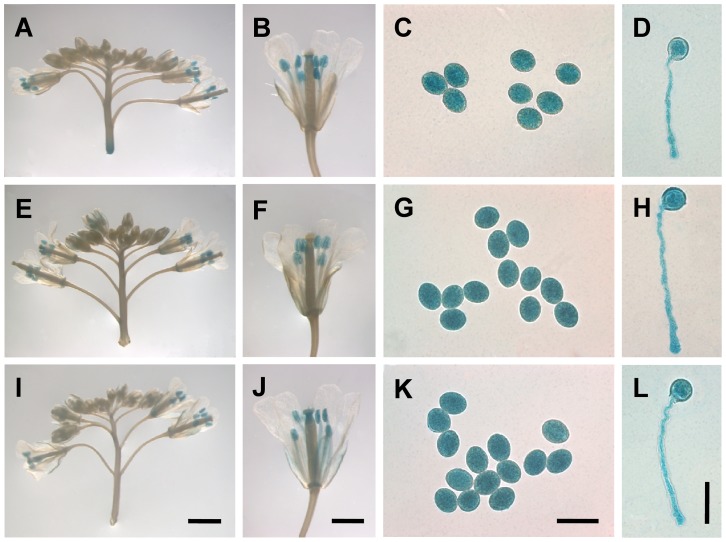
*MYB97*, *MYB101* and *MYB120* were expressed in pollen grains and pollen tubes. (**A**) to (**D**) Expression pattern of *MYB97*, as shown by GUS staining in an inflorescence (**A**), flower (**B**), mature pollen grains (**C**) and pollen tube (**D**). (**E**) to (**H**) Expression pattern of *MYB120*, as shown by GUS staining in an inflorescence (**E**), flower (**F**), mature pollen grains (**G**) and pollen tube (**H**). (**I**) to (**L**) Expression pattern of *MYB101*, as shown by GUS staining in an inflorescence (**I**), flower (**J**), mature pollen grains (**K**) and pollen tube (**L**). Bars = 2 mm in (**A**), (**E**) and (**I**); 1 mm in (**B**), (**F**) and (**J**) and 40 µm in (**C**), (**D**), (**G**), (**H**), (**K**), (**L**).

To investigate the expression patterns of these genes in detail, the promoters of *MYB97*, *MYB101* and *MYB120* (the genes in the first subgroup) were fused to a *GUS* reporter gene and introduced into wild type plants, respectively. GUS activity was detected mainly in the mature pollen grains and pollen tubes in the transgenic lines (Figure 2), consistent with the results of the RT-PCR and qRT-PCR assays. These results further demonstrated that the three MYB transcription factors are expressed mostly in mature pollen grains and pollen tubes, indicating that they could regulate transcription in the later development of male gametophytes.

### The MYB proteins exhibit highly similar amino acid sequences

To investigate the phylogenetic relationships among the seven MYB proteins, we generated a phylogenetic tree using DNAMAN software to analyze the amino acid sequences of the seven proteins. As shown in Figure 1A, the seven proteins were assigned into three main branches, consistent with their expression pattern (RT-PCR)-based classification. In particular, *MYB97*, *MYB101* and *MYB120*, which share 32% amino sequence identity, comprised one branch; *MYB33* and *MYB65*, which share a 62% amino acid identity, comprised the second branch; and *MYB81* and *MYB104*, which share 53% amino acid identity, comprised the third branch (Figure 1A). Recently, *MYB33* and *MYB65*, which have similar expression patterns, have been reported to be functionally redundant in anther development [29]. *MYB97*, *MYB101* and *MYB120* also have similar expression patterns in mature pollen grains and pollen tubes, implying that they may also be functionally redundant with each other. Therefore, this finding prompted us to study further their roles in pollen and pollen tubes by characterizing the *myb97 myb101 myb120* triple mutant.

### The *myb97 myb101 myb120* triple mutant exhibits drastically reduced fertility

**Figure 3:**
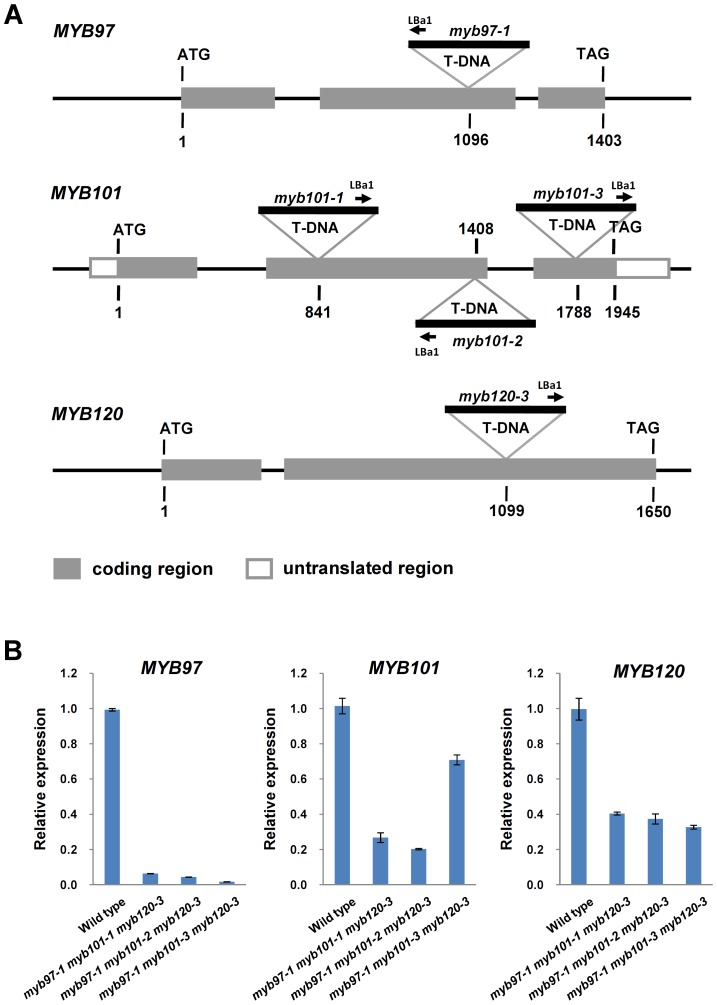
Molecular characterization of *myb97*, *myb101* and *myb120* mutants. (**A**) Schematic diagrams of the *MYB* gene structures and T-DNA insertion sites in the mutants. The gray and white boxes indicate the translated and untranslated regions, respectively. (**B**) The expression levels of *MYB97*, *MYB101* and *MYB120* genes in wild type (WT) and the triple homozygous mutants, as revealed by qRT-PCR.

To investigate their roles in the later development of male gametophytes, T-DNA insertion mutants of *MYB97*, *MYB101* and *MYB120* were purchased from the Arabidopsis Biological Resource Center (ABRC, www.arabidopsis.org) and backcrossed with wild type Col plants. Finally, we obtained single T-DNA insertion mutants for *MYB97* and *MYB120* (*myb97-1* and *myb120-3*, respectively). In the *myb97-1* mutant, the T-DNA insertion is located in the second exon of *MYB97*, 1096 bp downstream of the ATG start codon (Figure 3A). In the *myb120-3* mutant, the T-DNA is inserted in the second exon of *MYB120*, 1099 bp downstream of the ATG start codon (Figure 3A). Three single T-DNA insertion alleles were obtained for *MYB101*; the T-DNAs were inserted in the second and third exons, 841 bp, 1408 bp and 1788 bp downstream of the ATG start codon, respectively. These alleles were named *myb101-1*, *myb101-2* and *myb101-3*, respectively (Figure 3A).

For the further phenotypic characterization of these mutations, single homozygous mutant plants were generated by self-pollination. RT-PCR assays using mature pollen cDNA pools showed that the RNAs corresponding to *MYB97*, *MYB101* and *MYB120* were not detected in the corresponding single mutants. Further qRT-PCR assays also showed that the expression levels of the three genes were significantly decreased in the corresponding mutants compared with wild type plants (Figure S1). These results indicated that the T-DNA insertions had a strong impact on the expression of the corresponding genes. However, none of the single homozygous mutants exhibited any phenotypic defects (Table S3).

To investigate whether *MYB97*, *MYB101* and *MYB120* are functionally redundant with each other, we first generated double mutants of different allele combinations, namely *myb97-1 myb120-3*, *myb97-1 myb101-1*, *myb97-1 myb101-2*, *myb97-1 myb101-3*, *myb101-1 myb120-3*, *myb101-2 myb120-3* and *myb101-3 myb120-3*. The individual double homozygous mutants were identified by PCR-aided genotyping in the F2 generations of the crosses. None of the double mutants exhibited any defective phenotype of either the pollen or the vegetative parts, indicating that mutations in any two members of the subgroup did not affect male gametophyte development or fertility, similar to the phenotypes of the single mutations (Table S3). Then, we further generated triple mutants using these double mutant lines. Three triple-mutant lines with different allele combinations, namely *myb97-1 myb101-1 myb120-3*, *myb97-1 myb101-2 myb120-3* and *myb97-1 myb101-3 myb120-3*, were isolated. All of these triple mutants produced shorter siliques with lower seed sets compared to those of wild-type plants, whereas their vegetative parts appeared indistinguishable from those of wild type plants (Figure S2A). In the shorter siliques of the triple mutants, the seed set was drastically reduced, and many unfertilized ovules were found (Figure S3). The seed sets in the single and double mutants were almost identical to those of the wild type, i.e., nearly 100%. By contrast, the seed sets in the triple mutants were 38.93±6.13%, 36.61±6.13% and 72.75±6.13% of wild type, respectively (Table S3). These data suggested that *MYB97*, *MYB101* and *MYB120* are functionally redundant and are required for fertility in *Arabidopsis*.

### Expression of any single member from each subgroup of MYB proteins is sufficient to rescue the fertility-defective phenotype of the *myb97 myb101 myb120* triple mutants

To confirm that the phenotype of the *myb97 myb101 myb120* triple mutants was caused by the combination of mutations in *MYB97*, *MYB101* and *MYB120*, we investigated the expression levels of the three genes in the triple homozygous mutants and wild type by RT-PCR and qRT-PCR of the RNAs extracted from mature pollen grains of the triple homozygous mutants and wild type plants. The results showed that the expression of the three genes was significantly reduced in the triple mutant pollen grains compared to the wild type pollen grains (Figure 3B and S2B), suggesting that the triple mutant is a loss-of-function mutant.

**Figure 4:**
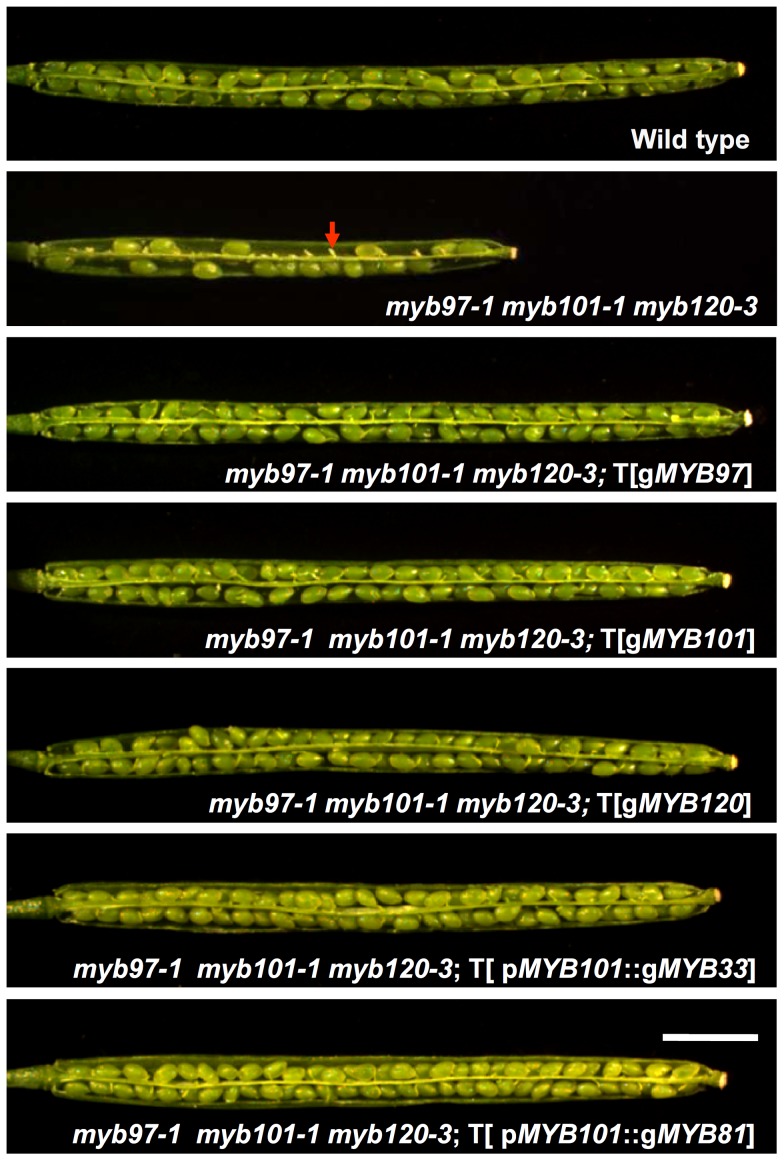
Complementation of the *myb97-1 myb101-1 myb120-3* mutant. The fertility of the *myb97-1 myb101-1 myb120-3* triple mutant was restored completely by transformation with *MYB97*, *MYB101*, *MYB120*, p*MYB101*::*MYB33* and p*MYB101*::*MYB81* complementation constructs. The red arrow indicates the unfertilized ovules. Bars = 2 mm.

Then, complementation experiments were performed using individual wild type genomic DNA fragments of *MYB97*, *MYB101* and *MYB120* (i.e., members of the first subgroup). The full-length genomic DNA fragments, including the predicted promoters, transcribed regions and 3′-end non-transcribed regions, were cloned and introduced into two independent triple homozygous mutant lines (*myb97-1 myb101-1 myb120-3* and *myb97-1 myb101-2 myb120-3*). Complementation screens in the *myb97-1 myb101-1 myb120-3* background yielded a total of 103 independent T1 transgenic lines (35 with the *MYB97* complementation construct, 23 with the *MYB101* complementation construct and 45 with the *MYB120* complementation construct). Preliminary observation showed that the seed set of the T1 plants was partially restored. Twenty T1 transgenic lines from each of the three different gene complementation constructs were selected for detailed examination. The quantitative data further confirmed that the phenotype of the siliques of *myb97-1 myb101-1 myb120-3* could be partially restored in the heterozygous transgenic MYB lines (data not shown). Then, homozygous transgenic MYB lines were generated by self-pollination for five randomly selected independent T1 lines for each complementation construct. In these T3 homozygous lines, the seed set was restored completely (Figure 4; Table 1). These results demonstrated that the fertility-defective phenotype of *myb97-1 myb101-1 myb120-3* could be restored by each of the single genes from the first subgroup (*MYB97*, *MYB101* and *MYB120*). Furthermore, *MYB97*, *MYB101* and *MYB120* also completely rescue the fertility-defective phenotype of *myb97-1 myb101-2 myb120-3* (Figure S4; Table S4).

**Table 1:** Complementation analysis of *myb97-1 myb101-1 myb120-3* triple homozygous mutant.

Genotypes	Seed set (%)
Wild type^a^	98.69±1.67
*myb97-1 myb101-1 myb120-3* a	33.05±7.05
*myb97-1 myb101-1 myb120-3*; T[*gMYB97*]^b^	99.14±1.64
*myb97-1 myb101-1 myb120-3*; T[*gMYB101*]^b^	99.50±1.21
*myb97-1 myb101-1 myb120-3*; T[*gMYB120*]^b^	97.41±2.87
*myb97-1 myb101-1 myb120-3*; T[*pMYB101::gMYB33*]^b^	93.18±3.58
*myb97-1 myb101-1 myb120-3*; T[*pMYB101::gMYB81*]^b^	97.56±2.27

The statistical analysis of the seed sets was performed in plants examined 50 days after transplantation into the soil.

a , 30 siliques were examined;

b , 75 siliques from five independent transgenic plants were examined.

T[*gMYB97*], transgenic *MYB97*; T[*gMYB101*], transgenic *MYB101*; T[*gMYB120*], transgenic *MYB120*; T[p*MYB101*::*gMYB33*], transgenic *MYB33*; T[p*MYB101*::*gMYB81*], transgenic *MYB81*.

In addition, we also performed an allelic analysis of the *myb101* alleles by generating three hybrid *myb101* allele combinations of the triple mutants, namely *myb97-1*/−;*myb101-1*/*myb101-2*;*myb120-3*/−, *myb97-1*/−;*myb101-1*/*myb101-3*;*myb120-3*/− and *myb97-1*/*−*;*myb101-2*/*myb101-3*;*myb120-3/−*. All three of these *myb101* allele hybrid triple mutants exhibited a fertility-defective phenotype similar to that of the triple mutants described above (Figure S3). The seed sets in the triple mutant siliques were 39.03±5.38%, 55.39±6.31% and 54.68±6.46%, respectively, of the wild type set (Table S3). These results demonstrated that the combination of mutations in *MYB97*, *MYB101* and *MYB120* caused a drastic reduction in fertility.

To investigate whether the MYB proteins in the other two subgroups had similar effects on fertility, the *MYB101* promoter was used to drive the expression of *MYB33*, from the second subgroup (*MYB33* and *MYB65*), and *MYB81*, from the third subgroup (*MYB81* and *MYB104*), in *myb97-1 myb101-1 myb120-3* homozygous mutants. The results showed that p*MYB101*::*MYB33* and p*MYB101*::*MYB81* were able to completely rescue the fertility-defective phenotype of *myb97-1 myb101-1 myb120-3* homozygous mutant (Figure 4; Table 1). Therefore, both *MYB33* and *MYB81* were able to perform the functions of *MYB97*, *MYB101* and *MYB120* in the first subgroup, indicating that the MYB proteins may have the same biochemical function with respect to fertility.

### The *myb97 myb101 myb120* triple mutants are male gametophyte-defective

To carry out further genetic analyses, we generated triple mutants that were homozygous for two mutations and heterozygous for the third mutation. For *myb97-1 myb101-1 myb120-3*, three independent combinations were obtained, namely *myb97-1*/+;*myb101-1*/−;*myb120-3*/−, *myb97-1*/−;*myb101-1*/−;*myb120-3/*+ and *myb97-1*/−;*myb101-1*/+;*myb120-3*/−. Because no obvious kanamycin-resistant selection marker was available for scoring the segregation ratio, PCR-aided genotyping was applied to identify the different genotypes of the progeny from the crosses using the triple mutants as male or female parents. In the self-pollination of *myb97-1 myb101-1 myb120-3*, the progeny of the mutant genotypes displayed a distorted segregation ratio of individuals with insertion and without insertion (Table 2). The segregation ratios were much lower than the typical Mendelian segregation ratio of 3 to 1, suggesting that the mutants were defective in gametophyte function. To determine the sex-related transmission efficiency (TE) of the mutations, reciprocal crosses between the mutants and wild type were performed. When the triple mutants were used as recipients (female) in crosses with wild-type pollen, approximately 50% of the resulting progeny contained the T-DNA insertions. When wild type plants were used as recipients in crosses with the triple mutant pollen grains, only a small number of the resulting progeny had the T-DNA insertions (Table 2). Furthermore, the analysis of *myb97-1 myb101-2 myb120-3* crosses also produced a similar result (Table S5).

**Table 2:** Genetic analysis of *myb97-1 myb101-1 myb120-3* heterozygous mutants.

Crosses (female×male)	W	Wo	W∶Wo	TE_F_ (%)	TE_M_ (%)
*myb97-1/+ myb101-1/*− *myb120-3/−* (self)	115	69	1.67∶1	NA	NA
+/+×*myb97-1/+ myb101-1/*− *myb120-3/−*	13	166	0.08∶1	NA	8
*myb97-1/+ myb101-1 myb120-3/−*×*+/+*	66	71	0.93∶1	93	NA
*myb97-1/− myb101-1/*− *myb120-3/+* (self)	108	63	1.71∶1	NA	NA
+/+×*myb97-1/− myb101-1/*− *myb120-3/+*	11	173	0.06∶1	NA	6
*myb97-1/− myb101-1/*− *myb120-3/+*×*+/+*	98	94	1.04∶1	104	NA
*myb97-1/− myb101-1/*+ *myb120-3/−* (self)	146	86	1.68∶1	NA	NA
+/+×*myb97-1/− myb101-1/+ myb120-3/−*	42	148	0.28∶1	NA	28
*myb97-1/− myb101-1/+ myb120-3/−*×*+/+*	94	98	0.96∶1	96	NA

W, with T-DNA; Wo, without T-DNA; TE, transmission efficiency = (W∶Wo)×100%; TE_F_, female transmission efficiency; TE_M_, male transmission efficiency; NA, not applicable; +/+, wild type; *myb97-1/−*, homozygous *myb97-1*; *myb97-1/+*, heterozygous *myb97-1*. The same format is used for *myb101-1* and *myb120-3*.

Reciprocal crosses were also performed between the *myb97-1 myb101-1 myb120-3* homozygous triple mutant and wild type plants. When wild type plants were manually self-pollinated or used as males in crosses with the *myb97-1 myb101-1 myb120-3* homozygous mutant, the resulting siliques were fully fertile with full seed sets. In contrast, when *myb97-1 myb101-1 myb120-3* homozygous mutants were manually self-pollinated or used as males in crosses with wild type plants, the resulting siliques were shorter and produced much lower seed sets (Table 3). In addition, for *myb97-1 myb101-2 myb120-3* and *myb97-1 myb101-3 myb120-3* homozygous mutants, the reciprocal crosses with wild type also produced similar results (Table 3).

**Table 3:** Genetic analysis of the *myb97 myb101 myb120* triple homozygous mutant.

Crosses (female×male)	Seed set (%)
Col×Col	94.64±3.43
Col×*myb97-1 myb101-1 myb120-3*	33.95±6.19
*myb97-1 myb101-1 myb120-3*×Col	93.37±5.50
*myb97-1 myb101-1 myb120-3* (self)	32.81±7.70
Col×Col	94.57±3.71
Col×*myb97-1 myb101-2 myb120-3*	25.48±5.03
*myb97-1 myb101-2 myb120-3*×Col	97.62±2.00
*myb97-1 myb101-2 myb120-3* (self)	29.45±5.85
Col×Col	92.70±5.29
Col×*myb97-1 myb101-3 myb120-3*	54.12±10.09
*myb97-1 myb101-3 myb120-3*×Col	90.67±5.00
*myb97-1 myb101-3 myb120-3* (self)	58.27±7.73

The statistical analysis of the seed sets was performed in plants examined 50 days after transplantation into the soil; 30 siliques were examined for each hybrid combination.

In summary, the triple mutants exhibited normal transmission through the female gametophytes but significantly reduced transmission through the male gametophytes. These results demonstrated that the *myb97 myb101 myb120* triple mutants are male gametophyte-defective.

### The *myb97 myb101 myb120* triple mutant pollen tubes fail to discharge sperm cells into the embryo sac

**Figure 5:**
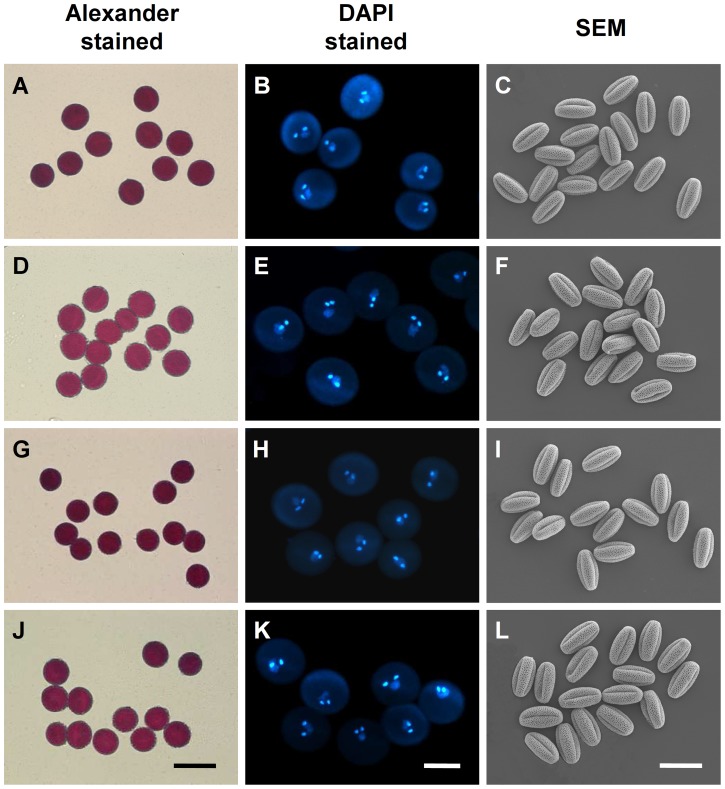
The *myb97 myb101 myb120* triple mutations did not affect the formation of pollen grains. The pollen grains were examined by Alexander staining, DAPI staining and scanning electron microscopy (SEM). (**A**) to (**C**) WT pollen grains. (**D**) to (**F**) *myb97-1 myb101-1 myb120-3* pollen grains. (**G**) to (**I**) *myb97-1 myb101-2 myb120-3* pollen grains. (**J**) to (**L**) *myb97-1 myb101-3 myb120-3* pollen grains. Bars = 40 µm in (**A**), (**D**), (**G**) and (**J**); 20 µm in (**B**), (**E**), (**H**) and (**K**); and 30 µm in (**C**), (**F**), (**I**) and (**L**).

To investigate the causes of the lower transmission of triple mutants through male gametophytes, we first examined the mature pollen grains. Alexander staining showed that all pollen grains from the triple mutants were indistinguishable from wild type pollen grains (Figure 5A, 5D, 5G and 5J). DAPI staining showed that the pollen grains from the triple mutant had two sperm cell nuclei and a vegetative nucleus, similar to the wild type pollen grains (Figure 5B, 5E, 5H and 5K). Scanning electron microscope (SEM) observations revealed no obvious morphological defects in the triple mutant pollen grains compared with wild type pollen grains (Figure 5C, 5F, 5I and 5L). Therefore, the triple mutations did not affect pollen formation.

Because the triple mutants could produce normal mature pollen grains, we next examined the germination of the triple-mutant pollen grains *in vivo*. The germination rates of the pollen grains from the three allelic triple mutants described above were 83.00±2.69%, 86.29±1.75% and 84.58±4.99%, respectively, similar to the wild type value of 88.59±4.95%. Therefore, the triple mutations also did not affect pollen germination.

**Figure 6:**
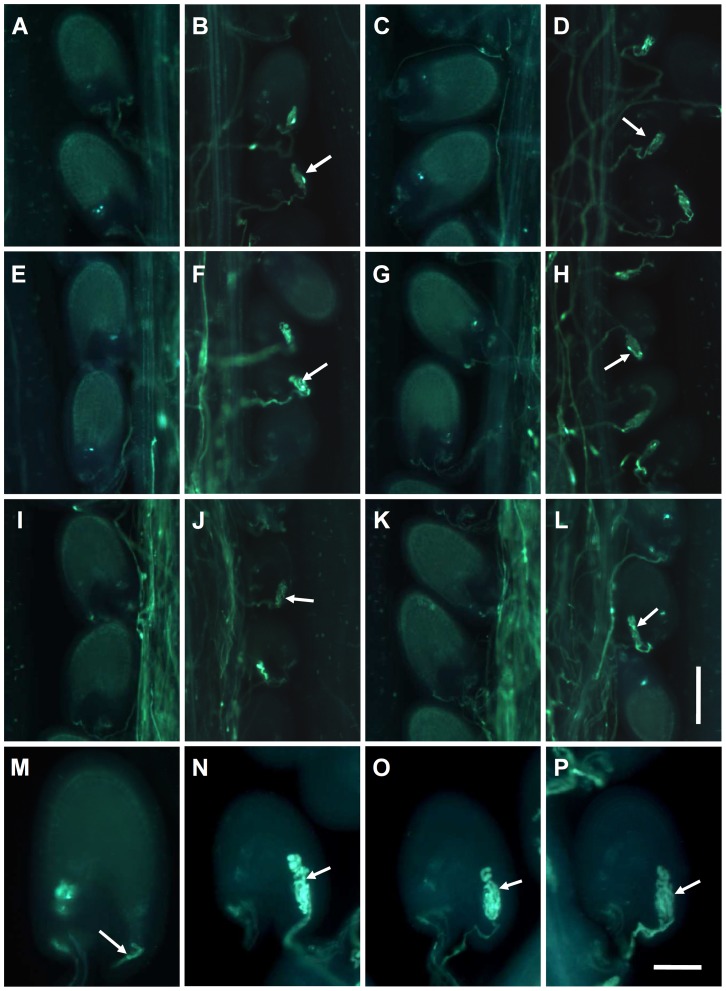
The *myb97 myb101 myb120* triple mutant pollen tubes did not stop growing and failed to discharge sperm cells into the embryo sacs. (**A**) to (**D**) The reciprocal crosses between *myb97-1 myb101-1 myb120-3* and wild type. (**A**) A Col silique pollinated with Col pollen. (**B**) A Col silique pollinated with *myb97-1 myb101-1 myb120-3* pollen. The white arrow indicates the overgrowing pollen tubes. (**C**) A *myb97-1 myb101-1 myb120-3* triple mutant silique pollinated with Col pollen. (**D**) A *myb97-1 myb101-1 myb120-3* triple mutant silique pollinated with *myb97-1 myb101-1 myb120-3* pollen. The white arrow indicates the overgrowing pollen tubes. (**E**) to (**H**) The reciprocal crosses between *myb97-1 myb101-2 myb120-3* and wild type. (**E**) A Col silique pollinated with Col pollen. (**F**) A Col silique pollinated with *myb97-1 myb101-2 myb120-3* pollen. The white arrow indicates overgrowing pollen tubes. (**G**) A *myb97-1 myb101-2 myb120-3* triple mutant silique pollinated with Col pollen. (**H**) *myb97-1 myb101-2 myb120-3* triple mutant silique pollinated with *myb97-1 myb101-2 myb120-3* pollen. The white arrow indicates overgrowing pollen tubes. (**I**) to (**L**) The reciprocal crosses between *myb97-1 myb101-3 myb120-3* and wild type. (**I**) A Col silique pollinated with Col pollen. (**J**) A Col silique pollinated with *myb97-1 myb101-3 myb120-3* pollen. The white arrow indicates overgrowing pollen tubes. (**K**) A *myb97-1 myb101-3 myb120-3* silique pollinated with Col pollen. (**L**) A *myb97-1 myb101-3 myb120-3* silique pollinated with *myb97-1 myb101-3 myb120-3* pollen. The white arrow indicates overgrowing pollen tubes. (**M**) to (**P**) Images showing the normal growth pattern of a wild type pollen tube (M) and the overgrowth patterns of *myb97-1 myb101-1 myb120-3* (**N**), *myb97-1 myb101-2 myb120-3* (**O**) and *myb97-1 myb101-3 myb120-3* (**P**) pollen tubes, as indicated by white arrows. Bars = 100 µm in (**A**) to (**L**); 40 µm in (**M**) to (**P**).

We then investigated growth of the triple mutant pollen tubes in pistils. At 4, 8 and 12 h after pollination (HAP), the pistils were harvested separately and examined by aniline blue staining to evaluate the pollen tube growth pattern. The results showed similar growth in the triple mutant and wild type pollen tubes, indicating that the triple mutations did not have a significant impact on growth of the pollen tubes prior to their encountering the female gametophyte. Surprisingly, however, unlike wild type, approximately 60% of the triple-mutant pollen tubes did not stop growing and failed to release the sperm cells into the embryo sacs at 48 HAP (Figure 6M–6P). The overgrowth of pollen tubes in the embryo sac resembled the phenotype of the *fer* mutant [13]–[15], which suggested that *MYB97*, *MYB101* and *MYB120* might take part in pollen tube reception. If this phenotype was the reason that the ovule could not be fertilized, then the percentage of the overgrowing pollen tubes must be consistent with that of the unfertilized ovule in the triple-mutant siliques. Then, we compared the rates of unfertilized ovules with the rate of pollen tube overgrowth in the triple mutant siliques. The results supported our hypothesis (Table 4). To confirm whether *MYB97*, *MYB101* and *MYB120* served as male components in pollen tube reception, reciprocal crosses were performed between the triple homozygous mutants and wild type to examine the growth of the triple-mutant pollen tubes in the wild type embryo sac. In the case when the wild type ovules were crossed with the triple mutant pollen, the percentage of overgrowing pollen tubes in the resulting pistils was close to that of the unfertilized ovules, similar to the data from the manual self-pollination of the triple mutants (Table 4; Figure 6). By contrast, very few overgrowing pollen tubes were observed in the crosses between triple mutant ovules and wild-type pollen tubes, similar to the data obtained in the manual self-pollination of wild type plant (Table 4; Figure 6). Therefore, the triple mutations affect the pollen tube reception through male gametophyte.

**Table 4:** Phenotypic characterization of the *myb97 myb101 myb120* triple mutants.

Crosses (female×male)	Normal pollen tubes (%)	Overgrowing pollen tubes (%)	Aborted ovules (%)
Col×Col	95.89±0.83	0.00±0.00	4.11±0.83
Col×*myb97-1 myb101-1 myb120-3*	35.16±2.87	60.65±2.63	4.19±3.55
*myb97-1 myb101-1 myb120-3*×Col	93.38±3.73	0.00±0.00	6.62±3.73
*myb97-1 myb101-1 myb120-3* (self)	34.31±1.74	60.89±2.41	4.80±2.62
Col×*myb97-1 myb101-2 myb120-3*	30.25±3.32	64.72±3.68	5.02±3.54
*myb97-1 myb101-2 myb120-3*×Col	95.01±3.57	0.29±0.72	4.70±3.44
*myb97-1 myb101-2 myb120-3* (self)	31.25±3.56	64.65±2.88	4.31±3.47
Col×*myb97-1 myb101-3 myb120-3*	64.74±5.46	27.72±5.35	7.54±2.17
*myb97-1 myb101-3 myb120-3*×Col	95.34±3.31	1.30±2.41	3.36±1.43
*myb97-1 myb101-3 myb120-3* (self)	60.76±3.99	35.54±5.96	3.71±3.29

More than 10 siliques were examined for each cross.

### The MYB proteins were localized to the nucleus and had transactivational activity in the yeast assay system

**Figure 7:**
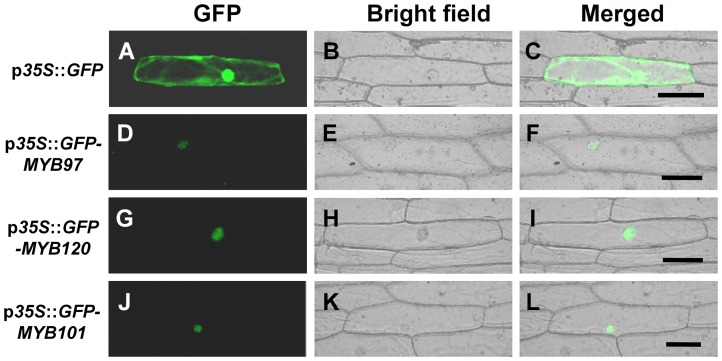
Subcellular localization of GFP-MYB fusion proteins. (**A**) to (**C**) Control, showing that the GFP signal from the p*35S*::*GFP* construct was distributed throughout the onion epidermal cell. (**D**) to (**F**) The GFP signal from the p*35S*::*GFP*-*MYB97* construct localizes to the nucleus. (**G**) to (**I**) The GFP signal from the p*35S*::*GFP*-*MYB120* construct localizes to the nucleus. (**J**) to (**L**) The GFP signal from the p*35S*::*GFP*-*MYB101* construct localizes to the nucleus. Bars = 1 mm in (**A**) to (**C**), (**G**) to (**I**); 100 µm in (**D**) to (**F**) and (**J**) to (**L**).

The MYB proteins belong to the R2R3 MYB transcription factor superfamily. They are expected to localize to the nucleus. To confirm the subcellular localization of MYB97, MYB101 and MYB120, N-terminal GFP fusion constructs of each of the MYB proteins driven by the 35S promoter were introduced into onion epidermal cells using particle bombardment. The observation showed that the fusion proteins were specifically localized to the nuclei (Figure 7D–7L), whereas the GFP control protein was found throughout the onion epidermal cells (Figure 7A–7C). These results were consistent with the predicted functions of the three MYB proteins as transcription factors.

**Figure 8:**
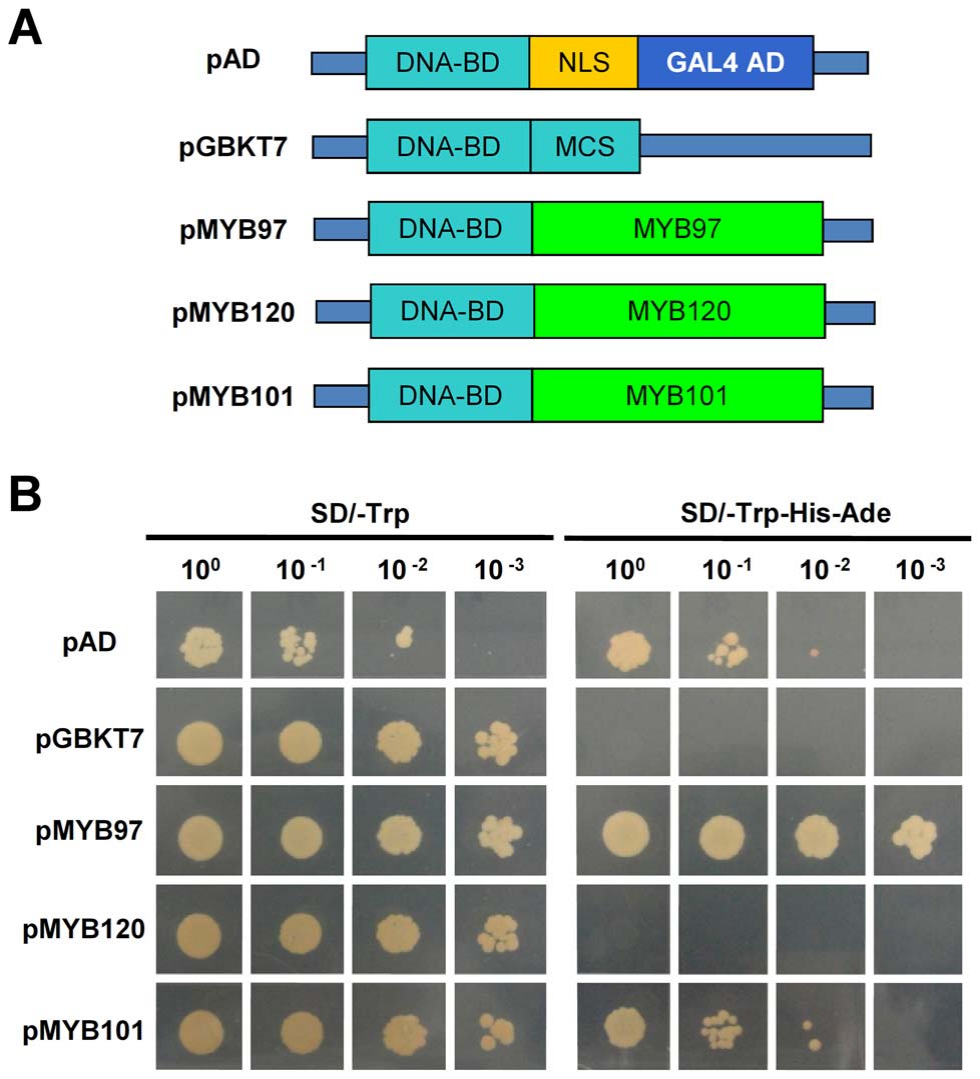
Transactivational activity assays. (**A**) Schematic diagrams of the different constructs used for transactivation activity assays. (**B**) The transactivation activity of MYB97 and MYB101 in yeast. DNA-BD, GAL4 DNA-binding domain; GAL4 AD, GAL4 activation domain; NLS, nuclear localization signal; MCS, multiple cloning site; MYB97, MYB101 and MYB120, the coding sequences (CDS) corresponding to *MYB97*, *MYB101* and *MYB120*.

The transcriptional activation ability of the three MYB proteins was investigated using a yeast assay system. Each of the three MYB proteins was fused to the GAL4 DNA binding domain in the pGBKT7 vector to generate the respective pMYB constructs (Figure 8A). Then, each of these plasmids, as well as the positive control pAD and negative control pGBKT7, were transformed into the yeast strain AH109. The growth patterns of the transformants were then examined (Figure 8B). The results showed that all transformants grew well on SD/-Trp medium. However, only the positive control pAD, pMYB97 and pMYB101 were able to grow on the selective SD/-Trp-His-Ade medium, whereas pMYB120 was not, indicating that MYB97 and MYB101 may function as transcription activators.

### The *myb97 myb101 myb120* triple mutation significantly affects the expression of downstream genes

As the first step to identify the downstream genes that are involved in pollen tube reception, we compared the gene transcription profile of *myb97-1 myb101-1 myb120-3* homozygous triple mutant pollen grains to that of wild type pollen grains by the microarray analysis using Affymetrix ATH1 Genome Arrays. Three biological replicates were applied in this study. The results of six chips were compared using the GeneSpring GX software (downloaded from http://www.genomics.agilent.com). Genes whose expression levels changed more than two-fold in mutant pollen grains compared with wild type pollen grains were selected as candidate target genes regulated by the three MYB transcription factors. Ultimately, 24 genes were selected based on this criterion; eight genes were obviously down-regulated, and the remaining 16 genes were significantly up-regulated in the triple-mutant pollen grains (Table S6). All 24 genes are expressed in pollen based on the data from TAIR (www.arabidopsis.org).

**Figure 9:**
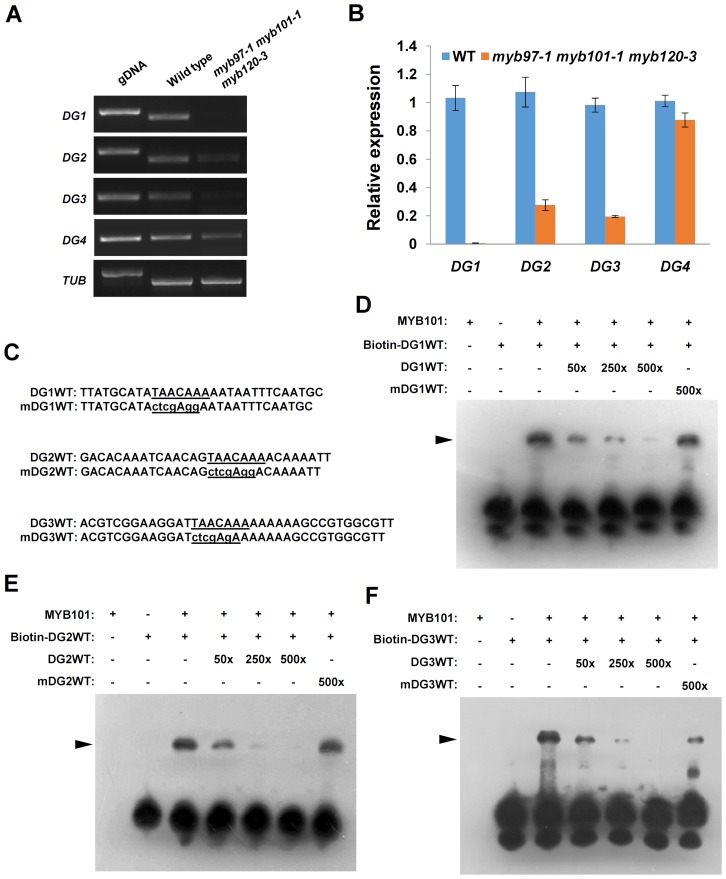
MYB101 binds to the MYBGAHV (TAACAAA) *cis*-element in the *DG1*, *DG2* and *DG3* promoters *in vitro*. (**A**) to (**B**) The expression of *DG1*, *DG2*, and *DG3* were significantly reduced in the *myb97 myb101 myb120* triple mutant, as revealed by RT-PCR (**A**) and qRT-PCR (**B**). (**C**) The sequences of the oligonucleotides used in the EMSA experiments. DG1WT, DG2WT and DG3WT are the wild-type versions of the MYBGAHV (TAACAAA) *cis*-elements (underlined) in the *DG1*, *DG2* and *DG3* promoters, respectively. The TAACAAA motifs were mutated as indicated by lowercase letters in the mDG1WT, mDG2WT and mDG3WT sequences. (**D**) MYB101 is able to bind to the MYBGAHV *cis*-element (TAACAAA) in the *DG1* promoter. Lanes 1 and 2 show reactions to which the MYB101 protein or the biotin-labeled DG1WT oligonucleotide was added, respectively. As shown in lane 3, a shift (black triangle) was observed when the MYB101 protein was added to the reaction containing the biotin-labeled DG1WT oligonucleotide. Lanes 4 to 7 show reactions in which unlabeled oligonucleotides were added to the binding reactions to compete with biotin-labeled DG1WT oligonucleotide. The competition becomes increasingly apparent with the unlabeled DG1WT oligonucleotide added at 50×, 250× and 500× molar excess in lanes 4, 5 and 6, respectively. Lane 7 shows that the unlabeled mDG1WT oligonucleotide competed only weakly, even at 500× molar excess. These results were reconfirmed in independent EMSA experiments. (**E**) MYB101 is able to bind to the MYBGAHV *cis*-element in the *DG2* promoter. The binding reaction containing the MYB101 protein and the biotin-labeled DG2WT oligonucleotide causes a clear shift. The unlabeled DG2WT oligonucleotide competed fully at 500× molar excess. No competition was observed when the unlabeled mDG2WT oligonucleotide was used at 500× molar excess. These results were reconfirmed in independent EMSA experiments. (**F**) MYB101 is able to bind to the MYBGAHV *cis*-element in the *DG3* promoter. The biotin-labeled DG3WT oligonucleotide was mixed with the MYB101 protein, and a shift was observed in the binding reaction. The unlabeled DG3WT oligonucleotide competed fully at 500× molar excess. Only weak competition was observed when unlabeled mDG3WT oligonucleotide was used at 500× molar excess. These results were reconfirmed in independent EMSA experiments.

To verify the results of the microarray data, we examined the expression levels of the eight down-regulated genes (DG) in the triple mutant and wild type further by RT-PCR and qRT-PCR. Three genes (*DG1*, *DG2* and *DG3*) were significantly down-regulated in the triple mutant relative to the wild type (Figure 9A and 9B), and selected for further analysis. It has been reported that the MYB gene from barley (*Hordeum vulgare*), *HvGAMYB*, a gene homologous to the MYB family, could bind to the TAACAAA motif of the barley high-pl α-amylase promoter and regulate the expression of α-amylase [30]–[32]. Therefore, the promoters of the three candidate genes were analyzed using the PLACE technique (http://www.dna.affrc.go.jp/PLACE/) for potential MYB-binding sites [33]. The results showed that TAACAAA motifs are present in the promoter regions of the three selected genes, implying that they might be direct targets of *MYB97*, *MYB101* and *MYB120*.

To investigate whether the MYB proteins were able to bind to the promoters of the three genes, electrophoretic mobility shift assays (EMSAs) were performed with the recombinant MYB domain of MYB101 and the three labeled oligonucleotides containing the TAACAAA sequence derived from the promoters of *DG1*, *DG2* and *DG3* (Figure 9C). As a control, the recombinant MYB101 protein and a labeled oligonucleotide were added alone to the native gel. The reaction of the two components caused a shift in the mobility of the labeled oligonucleotide, indicating that MYB101 was able to bind these oligonucleotides *in vitro* (Figure 9D–9F). To further determine whether the interaction is specific for the TAACAAA motif, unlabeled wild type and mutated oligonucleotides were added to the reaction as competitors. The interaction between MYB101 and a labeled oligonucleotide was clearly disrupted by the addition of excess wild type competitor but only weakly disrupted by the mutated competitor in which the TAACAAA sequence was mutated (Figure 9D–9F). These results demonstrated that the MYB101 protein could bind specifically to the TAACAAA-containing sequences derived from the promoters of *DG1*, *DG2* and *DG3*, which were down-regulated in the *myb97 myb101 myb120* triple mutants.

## Discussion

### *MYB97*, *MYB101* and *MYB120* function as novel male factors in pollen tube reception

We report here the identification and characterization of a group of MYB transcription factors that function as male factors involved in pollen tube reception. The final stage of later development of male gametophytes is pollen tube reception. After the pollen tube enters the embryo sac, it encounters and interacts with the receptive synergid. The interactive signaling process triggers the rupture of the pollen tube and the release of the sperm cells into the embryo sac [11], [12]. This interaction between male and female gametophytes is expected to involve components from both the male (pollen tube) and female (synergid) partners. To date, although several components in the female synergid have been identified to be involved in pollen tube reception in *Arabidopsis* and maize [15]–[17], [19], [22], [34], much less information about the male components involved in this process is available. AMC is expressed in both the pollen tube and synergid. The *amc* mutant also exhibits a pollen tube overgrowth phenotype when the mutant pollen tube enters the mutant embryo sac [21]. Therefore, it is unlikely to be a male-specific component of the pollen reception pathway. Plants with mutations in the pollen tube-expressed *ANX1*/*ANX2*, *ACA9* and *KZM1* genes exhibit phenotypes related to pollen tube growth but not the typical phenotype of pollen tube overgrowth [22]–[25]. More evidence is required to clarify whether these genes are actually involved in the pollen tube reception process. In this study, we demonstrate that at least three MYB members, MYB97, MYB101 and MYB120, are expressed in pollen but not in female partner synergids. The *myb97 myb101 myb120* triple mutants exhibited the typical pollen tube overgrowth phenotype, almost identical to those of *fer*, *lre* and *nta* mutants [11]–[20]. The overgrowth of the triple-mutant pollen tubes also occurred in a cross of wild type plants with the *myb97 myb101 myb120* triple mutant pollen grains, as expected. Therefore, we conclude that the MYB97, MYB101 and MYB120 proteins function as male factors to participate in pollen tube reception, which supports the previous hypothesis that the transcription factors play essential roles in pollen-pistil interactions [10]. This new finding will be useful in the further search for new components of the pollen tube reception pathway in the future.

Our data demonstrated that MYB97, MYB101 and MYB120 are localized to the nucleus of the onion epidermal cells and that at least MYB97 and MYB101 have transcription transactivation activity in a yeast assay system. In addition, the recent work showed that the three MYBs accumulated in the pollen tube nucleus during pollen tube growth through the pistil [35]. Therefore, these three proteins likely function as transcription factors and control pollen tube reception by regulating the expression of the downstream genes that are involved in the pollen tube reception process. Microarray analysis showed that the triple mutation affected the expression of at least 24 genes in mature pollen grains, among which eight were down-regulated and 16 were up-regulated, including transcription factor genes, secreted protein genes and the genes that may be involved in cell signaling. RT-PCR and qRT-PCR analysis further confirmed that at least *DG1*, *DG2* and *DG3* were regulated by MYB97, MYB101 and MYB120. The EMSA assays further demonstrated that recombinant MYB101 protein expressed in an *E. coli* system could bind to the promoter regions of the three *DG* genes *in vitro*. These data indicate that the MYB proteins may regulate the expression of downstream genes by binding to their promoter regions, as is the case for other MYB transcription factors [30]–[32]. However, whether these MYB-regulated downstream genes actually function in pollen tube reception remains unclear. Further investigation is required to address this question.

FER has been proposed to function as a signaling receptor in pollen tube reception [15]. This raises the important question of where the signal molecule or ligand comes from. The most likely answer is that this signal could come from the pollen tube. Namely, FER might be activated by a ligand or signaling molecule from the pollen tube and subsequently trigger a downstream signal transduction pathway to feed back to the pollen tube and cause pollen tube rupture [11], [12], [15], [34]. However, this ligand or signaling molecule has not yet been identified. Our results showed that the *myb97 myb101 myb120* triple mutation affected the expression of several pollen-expressed genes. This result raises another interesting question, i.e., whether there are any candidate FER ligands among the genes affected by the *myb97 myb101 myb120* triple mutation. We identified one gene, *DG3* (*AT3G19690*), which encodes a defensin-like protein with homologues that have been proposed to function as a signaling molecule in pollen tube guidance [36]. Our microarray, RT-PCR and qRT-PCR analyses showed that the expression of *DG3* at a transcriptional level was significantly reduced in *myb97-1 myb101-1 myb120-3* homozygous mature pollen grains compared to wild type mature pollen grains. Additionally, the expression level of *DG3* (*AT3G19690*) was changed from low to high during pollen tube growth [10]. Furthermore, EMSA assays suggested that MYB101 could bind to the TAACAAA motif-containing sequence derived from the promoter region of *AT3G19690*. However, we still lack direct experimental evidence that DG3 is a ligand of the FER receptor. Nevertheless, these findings will prompt future work toward the identification and characterization of potential FER receptor-associated signaling factors from the pollen tube.

### *MYB97*, *MYB101* and *MYB120* are functionally redundant in pollen tube reception

The functional redundancy of the genes involved in male gametophyte development has been noted in different transcription factor gene families, for example, between *MYB33* and *MYB65* in anther development and among the AtMIKC* genes in pollen germination and pollen tube growth [7], [23], [24], [29]. In this study, the MYB97, MYB101 and MYB120 proteins are highly similar to each other in amino acid sequence. Furthermore, only *myb97 myb101 myb120* triple mutants exhibited a pollen tube reception-defective phenotype; the double and single mutants did not. Moreover, the expression of a single gene from the MYB family could complement the phenotype of the triple mutant. In addition, RT-PCR and GUS analyses showed that *MYB97*, *MYB101* and *MYB120* are mainly expressed in mature pollen and pollen tubes, consistent with a role in pollen tube reception. These data demonstrate that the MYB proteins are functionally redundant as predicted previously [10].

In the *myb97 myb101 myb120* triple mutants, approximately 40% of the mutant pollen tubes were able to complete the process of sperm discharge, leading to successful fertilization and seed development, indicating that the triple mutation does not completely block the discharge of sperm cells. The reason for this observation remains unclear. Our results showed that p*MYB101*::*MYB33* and p*MYB101*::*MYB81* constructs could complement the phenotype of *myb97 myb101 myb120* triple mutants, suggesting that the MYB proteins function in pollen tube reception fertility. Thus, one possible explanation is that the other MYB members might contribute to this process. To address this question, we initiated the construction of higher-level multiple mutants or universal disruption of all the seven pollen-expressed MYB genes identified in this study. Because MYB33 and MYB65 were more closely related to MYB97, MYB120 and MYB101 than MYB81 and MYB104 (as shown in the phylogenetic tree in Figure 1A), mutants of these two genes were first used to construct quadruple mutations. The results showed that neither the *myb33 myb97 myb101 myb120* nor the *myb65 myb97 myb101 myb120* quadruple mutant exhibited a more severe phenotype than the triple mutant (Figure S5; Table S7), indicating that neither *MYB33* nor *MYB65* has a significant impact on pollen tube reception. This finding is consistent with their much lower expression in pollen (Figure 1C). Furthermore, as shown in Figure 1C, the expression levels of *MYB81* and *MYB104* are much lower than even that of *MYB33*. Thus, *MYB81* and *MYB104* are also unlikely to make significant contributions to pollen tube reception. Further investigation will be required to understand whether other MYB genes may be involved in pollen tube reception.

It is also worth noting that qRT-PCR assays using primer pairs targeting the sequences upstream of the T-DNA insertion sites showed that truncated MYB101 and MYB120 transcription products were present in the *myb97 myb101 myb120* triple mutants (Figure 3B). To date, however, we have not determined whether these truncated transcripts are associated with the partial fertility of the *myb97 myb101 myb120* triple mutant. Further investigation is required to address this question.

While this manuscript was under review, Leydon and coworkers [35] reported the characterization of the *myb97 myb101 myb120* triple mutant using a different approach. The study was more focused on cell biological characteristic of the triple mutant in pollen tube reception. The results clearly show that lacking expression of *MYB9*7, *MYB101* and *MYB120* caused defect in discharge of sperm cells and affected degeneration of the synergid cells. In comparison, our study had a more detailed genetic analysis. The results indicate that the MYB proteins from the MYB family (including MYB33, MYB65, MYB81, MYB97, MYB101, MYB104 and MYB120) are functionally redundant. Our results also demonstrate that the MYB proteins can bind to the promoter sequences of the genes whose expression was affected by the *myb97 myb101 myb120* triple mutation. In addition, our microarray assay was different from the one reported by Leydon and coworkers [35]. Our microarray assay with mature pollen grains showed that the expression of 24 genes were significantly changed in the *myb97 myb101 myb120* triple mutant pollen grains, among which eight were down-regulated and 16 were up-regulated. In the microarray analysis with the pistil 8 hr after pollination, reported by Leydon and coworkers [35], 48 genes with significantly different transcript abundance were identified among which 45 were down-regulated and three were up-regulated [35]. Comparison of the data from the two assays showed that none of the above genes was found to be affected overlappingly in the both microarray assays, implying that the impact of the MYBs on gene expression in mature pollen grains could be different from that in the growing pollen tubes. Nevertheless, more studies are required to address whether these genes are actually involved in pollen tube reception.

In conclusion, our results provide important new evidence to demonstrate that male components participate in pollen tube reception and help us to understand further the mechanism of male-female communication during pollination and fertilization.

## Materials and Methods

### Plant materials and growth conditions

All mutant seeds used in this study were obtained from the *Arabidopsis* Biological Resource Center (ABRC, www.arabidopsis.org): *myb97-1* (Salk_112329C), *myb101-1* (Salk_061355), *myb101-2* (Salk_149918C), *myb101-3* (Salk_039489C), and *myb120-3* (Salk_063698). The mutant and wild type seeds were first plated on Murashige and Skoog (MS) [37] agar plates supplemented with or without 25 mg/L hygromycin to select for transgenic lines. After two days at 4°C, the plates were transferred to the growth chambers with a 16 h light/8 h dark cycle at 22°C. Then, the seedlings were transplanted into soil ten days after germination and grown under the same conditions as that used for seed germination.

### Identification of T-DNA insertion mutants

The T-DNA insertion sites were confirmed by PCR using the T-DNA–specific primer LBa1 paired with the gene-specific primers MYB97-1-RP, MYB101-1-RP, MYB101-2-RP, MYB101-3-RP and MYB120-3-RP. The homozygous mutant plants were selected by PCR using these RP primers paired with the following LP primers: MYB97-1-LP, MYB101-1-LP, MYB101-2-LP, MYB101-3-LP and MYB120-3-LP. All primers used in this work are listed in Table S8.

### RT-PCR and qRT-PCR analyses

Total RNA samples were extracted from different *Arabidopsis* tissues using a total RNA extraction kit (Bioteke, Beijing, China). First-strand cDNA was synthesized using the alfalfa mosaic virus reverse transcriptase kit (TaKaRa, Dalian, China) according to the supplier's instructions. *Tubulin* was used as an internal control for RT-PCR. The primer pairs used in the assays were TUB-RT-F/TUB-RT-R, MYB97-RT-F/MYB97-RT-R, MYB101-RT-F/MYB101-RT-R, MYB120-RT-F/MYB120-RT-R MYB33-RT-F/MYB33-RT-R, MYB65-RT-F/MYB65-RT-R, MYB81-RT-F/MYB81-RT-R and MYB104-RT-F/MYB104-RT-R. The cDNA pools from different tissues were used for the expression pattern analyses, while the cDNA pools from mature pollen were used for comparisons of the gene expression levels in the mutant and wild type plants. Amplifications were run for 35 cycles for *TUB*, *MYB33*, *MYB65*, *MYB97*, *MYB101* and *MYB104* and for 45 cycles for *MYB81* and *MYB120*.

The RNA samples used in the qRT-PCR analysis were extracted from mature pollen grains. Then, cDNAs were reverse transcribed using SuperScript III Reverse Transcriptase (Invitrogen, 18064-014, USA) and random primers (Promega, Madison, USA). qRT-PCR assays were performed using Power SYBR Green PCR Master Mix and the Applied Biosystems 7500 Real-Time PCR System (Applied Biosystems, http://www. appliedbiosystems.com). The experiments were repeated three times. The *ACTIN2* RNA levels were quantified as an internal control to normalize the RNA quantity. The primer pairs used were ACTIN2-rl-F/ACTIN2-rl-R, MYB97-rl-F/MYB97-rl-R, MYB101-rl-F/MYB101-rl-R, MYB120-rl-F/MYB120-rl-R, MYB33-rl-F/MYB33-rl-R, MYB65-rl-F/MYB65-rl-R, MYB81-rl-F/MYB81-rl-R, MYB104-rl-F/MYB104-rl-R, DG1-rl-F/DG1-rl-R, DG2-rl-F/DG2-rl-R, DG3-rl-F/DG3-rl-R and DG4-rl-F/DG4-rl-R. The thermocycling settings were as follows: 10 min at 95°C (one cycle) followed by 15 s at 95°C and 34 s at 60°C (40 cycles). After each run, a dissociation curve was acquired by heating the samples from 60 to 95°C to ensure amplification specificity.

### Phenotypic characterization of siliques and mature pollen grains

For observations of unfertilized ovules, we dissected the siliques with a needle and scored the unfertilized ovules under a dissecting microscope. Alexander staining of mature pollen grains was performed as described previously [38]. DAPI staining of pollen grains was performed as described by Xia et al. [39]. Morphological observations of pollen grains by SEM were performed as described by Jiang et al. [40].

### Aniline blue staining of germinated pollen grains and pollen tubes *in vivo*

Flowers were emasculated before anthesis and pollinated with the pollen grains from wild type and mutants. To score pollen germination rates, pistils that were pollinated with a limited number of pollen grains were harvested at 1–2 h after pollination, stained in aniline blue buffer for 15 min and examined under a fluorescence microscope, as described previously [40]. For the phenotypic analysis of pollen tubes, the pistils were harvested 48 h after pollination and fixed in an acetic acid/ethanol solution (1∶3 [v/v]) for 2 h. The fixed pistils were then softened in an 8 M NaOH solution overnight. The pistils were washed several times with clean water and subsequently stained with aniline blue for 5 h in the dark. The aniline blue buffer contained 0.1% aniline blue in 0.1 M K_2_HPO_4_-KOH buffer, pH 11.0. The pollen tubes in the stained pistils were observed using a Leica DM2500 microscope (Leica, Wetzlar, Germany).

### GUS assay

Different transgenic plant tissues were stained in 100 mM NaPO_4_ (pH 7.0) solution containing 0.5 mM potassium ferricyanide [K_3_Fe(CN)_6_], 0.5 mM potassium ferrocyanide [K_4_Fe(CN)_6_], 0.1% Triton X-100, 10 mM EDTA and 0.5 mg/ml bromochloroindoyl-β-glucuronide [40]. The tissues were stained at 37°C for 2–3 h and then clarified in an acetic acid/ethanol solution (1∶3 [v/v]) overnight. The GUS-stained tissues were then examined using a Leica DM2500 microscope equipped with DIC system and MZ10F stereo microscope (Leica, Wetzlar, Germany).

### Molecular cloning

For the gene expression pattern assays, promoter fragments of the *MYB97*, *MYB101* and *MYB120* genes were amplified by PCR using the following gene-specific primer pairs: MYB97-GUS-F/MYB97-GUS-R, MYB101-GUS-F/MYB101-GUS-R and MYB120-GUS-F/MYB120-GUS-R. The resulting fragments were subcloned upstream of the *GUS* reporter gene in the pCAMBIA1300 vector (CAMBIA, Australia) and introduced into wild type plants using the *Agrobacterium*-mediated infiltration method [41].

For complementation experiments, full-length genomic DNA fragments of *MYB97*, *MYB101* and *MYB120*, including the predicted promoters, transcribed regions and 3′-end non-transcribed regions, were amplified by PCR using the following primer pairs: MYB97-F-1F/MYB97-F-1R and MYB97-F-2F/MYB97-F-2R for MYB97, MYB101-F-1F/MYB101-F-1R and MYB101-F-2F/MYB101-F-2R for MYB101, and MYB120-F-1F/MYB120-F-1R and MYB120-F-2F/MYB120-F-2R for MYB120. The resulting fragments were cloned into a pMD-18 T vector for sequence validation. Then, the full-length genomic DNA fragments were subcloned into the pCAMBIA1300 vector and introduced into the *myb97-1 myb101-1 myb120-3* and *myb97-1 myb101-2 myb120-3* triple homozygous mutants as described above. For *MYB33* and *MYB81* complementation assays, the transcribed regions of *MYB33* and *MYB81* were amplified by PCR using the following primer pairs: MYB33-T-F/MYB33-T-R and MYB81-T-F/MYB81-T-R. The resulting fragments were subcloned into the pCAMBIA1300 vector to generate p*MYB101*::*MYB33* and p*MYB101*::*MYB81* constructs and then introduced into the *myb97-1 myb101-1 myb120-3* triple homozygous mutant as described above.

To evaluate the subcellular localization of the MYB97, MYB101 and MYB120 proteins, the CDS fragments of *MYB97* and *MYB101* and the genomic transcribed region of *MYB120* were amplified by PCR using the following primer pairs: MYB97-G-F/MYB97-G-R, MYB101-G-F/MYB101-G-R and MYB120-G-F/MYB120-G-R. Then, the resulting fragments were subcloned into the pCAMBIA1300 vector to generate N-terminal fusions of the genes with the *GFP* coding sequence driven by the 35S promoter. The fusion expression vectors were introduced into onion epidermal cells by particle bombardment as described by Zhu et al. [42].

### Transactivational activity assay

For transactivational activity assays, the CDSs of the *MYB97*, *MYB101* and *MYB120* genes were amplified by PCR using the following primer pairs: MYB97-BD-F/MYB97-G-R, MYB101-BD-F/MYB101-G-R and MYB120-BD-F/MYB120-G-R. Then, the fragments were fused to the GAL4 DNA binding domain in the pGBKT7 vector to generate pMYB constructs. For the positive control pAD, the AD fragment from the pGADT7 vector was amplified by PCR using the primer pair AD-F/AD-R and subcloned into the pGBKT7 vector. The resulting plasmids were transformed into the yeast strain AH109, and their transactivational activities were examined.

### Microarray analysis

The RNA samples used for the microarray assays were extracted from mature pollen grains of wild type and *myb97-1 myb101-1 myb120-3* homozygous mutants. ATH1 Genome Arrays were used to compare the transcriptomes of wild type and *myb97-1 myb101-1 myb120-3* homozygous mutant pollen grains. Three biological replicates were performed. Raw data (CEL files) for six ATH1 chips were analyzed using the GeneSpring GX software (downloaded from http://www.genomics.agilent.com/). Genes that displayed greater than two-fold changes in expression were selected as candidates for regulation by the three transcription factors.

### Expression and purification of the MYB101 protein and electrophoretic mobility shift assay (EMSA)

The cDNA fragment of the MYB domain (amino acid 1 to 133) of MYB101 was amplified using the primer pair MYB101-P-F/MYB101-P-R and cloned into the *Bam*H I-*Sal* I site of pET-30a(+) (Novagen, Madison, USA). The resulting construct was introduced into *Escherichia coli* strain BL21 (DE3) pLysS (TIANGEN, China) to generate a 6×His-MYB101^1–133^ fusion protein. The expression of the fused protein was induced by 1 mM isopropyl β–D-1-thiogalactopyranoside (IPTG) at 18°C for 16 h. The recombinant protein was purified using PrepEase His-Tagged Protein Purification Kit (USB, 78793, Germany) and dialyzed against storage buffer (20% glycerol, 10 mM Tris-Cl, 50 mM KCl, 1 mM DTT, pH 8.0).

The EMSA was performed using the LightShift Chemiluminescent EMSA Kit (Pierce, 20148, USA) according to the instructions provided by the supplier. The 5′-biotin-modified oligonucleotides were synthesized by Invitrogen (Invitrogen, China). To generate the double-stranded oligonucleotides, complementary pairs of oligonucleotides were annealed in a Tris buffer (10 mM Tris-Cl, 1 mM EDTA, 50 mM NaCl, pH 8.0) in a thermocycler (step 1: 95°C for 5 min; step 2: 95°C (−1°C/cycle) for 1 min; 70 cycles). The notation “−1°C/cycle” indicates that the temperature of the heating block was decreased by 1°C per cycle. The binding reactions were carried out in binding buffer (10 mM Tris-Cl, 50 mM KCl, 1 mM DTT, pH7.5) at room temperature for 20 min. For the reactions shown in lanes 2 to 7, 20 fmol of biotin-labeled oligonucleotides, 200 ng of recombinant protein, 50 ng poly(dI·dC) and an appropriate concentration of unlabeled oligonucleotides were mixed in a 20-µl binding reaction. The reaction shown in lane 1 contained no protein, as a control. The reaction products were separated in 1-mm-thick 6% native polyacrylamide gels in 0.5× TBE buffer (45 mM Tris base, 45 mM boric acid and 1 mM EDTA, pH 8.0, precooled to 4°C) at 100 V for 1 h. The oligonucleotides were transferred to Amersham Hybond-N^+^ nylon membranes (GE, RPN303B, UK) by electroblotting at 380 mA for 30 min in 0.5× TBE and then cross-linked with a BLX-254 UV crosslinker (Vilber Lourmat, France). The biotin-labeled oligonucleotides were detected according to the instructions provided with the Chemiluminescent Nucleic Acid Detection Module (Pierce, 89880, USA).

## Supporting Information

Figure S1 The *myb97*, *myb101* and *myb120* mutations disrupted the expression of the *MYB* genes in pollen grains, as revealed by RT-PCR and qRT-PCR. (A) The reduced expression of *MYB97* in the *myb97-1* mutant. (B) The reduced expression of *MYB101* in the *myb101-1*, *myb101-2* and *myb101-3* mutants, respectively. (C) The reduced expression of *MYB120* in the *myb120-3* mutant.
(TIF)

Figure S2 Isolation and characterization of the *myb97 myb101 myb120* triple mutants. (A) The vegetative growth of the *myb97 myb101 myb120* triple mutants is normal. (B) The transcript of *MYB97*, *MYB101* and *MYB120* in wild type and triple mutants, revealed by RT-PCR.
(TIF)

Figure S3 Phenotypic characterization and genetic analysis of the *myb97 myb101 myb120* triple mutants. The triple *myb97 myb101 myb120* mutants produced shorter siliques with reduced seed set compared to that of wild type. The red arrows indicate the unfertilized ovules. Bars = 2 mm.
(TIF)

Figure S4 Complementation analysis of myb97-1 *myb101-2 myb120-3* triple mutant by *MYB97, MYB101* and *MYB120* genomic DNAs. Transformation of *MYB97, MYB101* and *MYB120* complementation constructs could restore the fertility of the *myb97-1 myb101-2 myb120-3* triple mutants completely. The red arrows indicate the unfertilized ovules. Bars =2 mm.
(TIF)

Figure S5 Phenotypic characterization of the quadruple *myb* mutants. The quadruple *myb* mutants did not exhibit more severe phenotypes compared to that of *myb97-1 myb101-1 myb120-3* triple mutant. The red arrows indicate the unfertilized ovules. Bars = 2 mm.
(TIF)

Table S1 The MYB transcription factors involved in male gametophyte development in *Arabidopsis*.
(DOCX)

Table S2 Identification of the MYBs.
(DOCX)

Table S3 The *myb97 myb101 myb120* triple mutations reduced fertility. The statistics of silique length and seed set was performed in plants examined 50 days after transplantation into the soil; 30 siliques were examined for each combination.
(DOCX)

Table S4 Complementation analysis of the *myb97-1 myb101-2 myb120-3* homozygous mutant. The statistics of silique length and seed set was performed in plants examined 50 days after transplantation into the soil. a, 30 siliques were examined; b, 75 siliques from 5 independent transgenic plants were examined. T[*gMYB97*], transgenic *MYB97*; T[*gMYB101*], transgenic *MYB101*; T[*gMYB120*], transgenic *MYB120*.
(DOCX)

Table S5 Genetic analysis of the *myb97-1 myb101-2 myb120-3* heterozygous mutant. W, with T-DNA; Wo, without T-DNA; TE, transmission efficiency: (W∶Wo)×100%; TE_F_, female transmission efficiency; TE_M_, male transmission efficiency; NA, not applicable; +/+, wild type; *myb97-1/−*, homozygous *myb97-1*; *myb97-1/+*, heterozygous *myb97-1*; The same format is used for *myb101-1* and *myb120-3*.
(DOCX)

Table S6 The genes whose expression was affected in the *myb97-1 myb101-1 myb120-3* triple mutant, revealed by microarray analysis. Fold change = Log_2_(mutant/WT).
(DOCX)

Table S7 Phenotypic analysis of the quadruple *myb* mutants. The statistics of silique length and seed set was performed in plants examined 50 days after transplantation into the soil; 30 siliques were examined for each combination.
(DOCX)

Table S8 Sequences of the primers used in this study.
(DOCX)

Text S1 Supplemental references.
(DOCX)
