# Supplementary figures and images for: MYB97, MYB101 and MYB120 Function as Male Factors That Control Pollen Tube-Synergid Interaction in Arabidopsis thaliana Fertilization
Source: PLoS Genet. 2013 Nov 21;9(11):e1003933. doi: 10.1371/journal.pgen.1003933 (PMC3836714; doi:10.1371/journal.pgen.1003933)

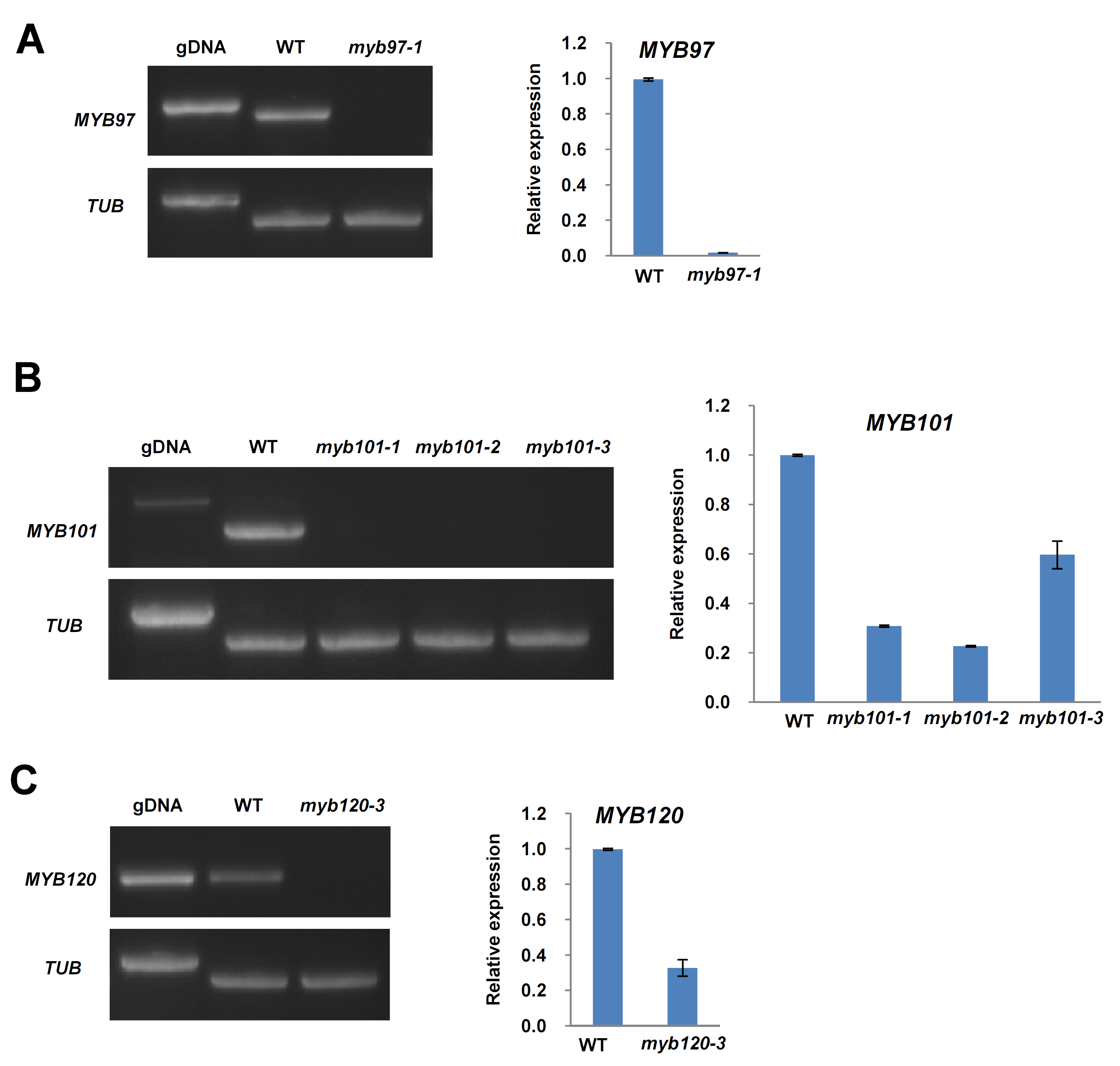

Supplement: Figure S1 — The myb97, myb101 and myb120 mutations disrupted the expression of the MYB genes in pollen grains, as revealed by RT-PCR and qRT-PCR. (A) The reduced expression of MYB97 in the myb97-1 mutant. (B) The reduced expression of MYB101 in the myb101-1, myb101-2 and myb101-3 mutants, respectively. (C) The reduced expression of MYB120 in the myb120-3 mutant. (TIF) [file pgen.1003933.s001.tif]

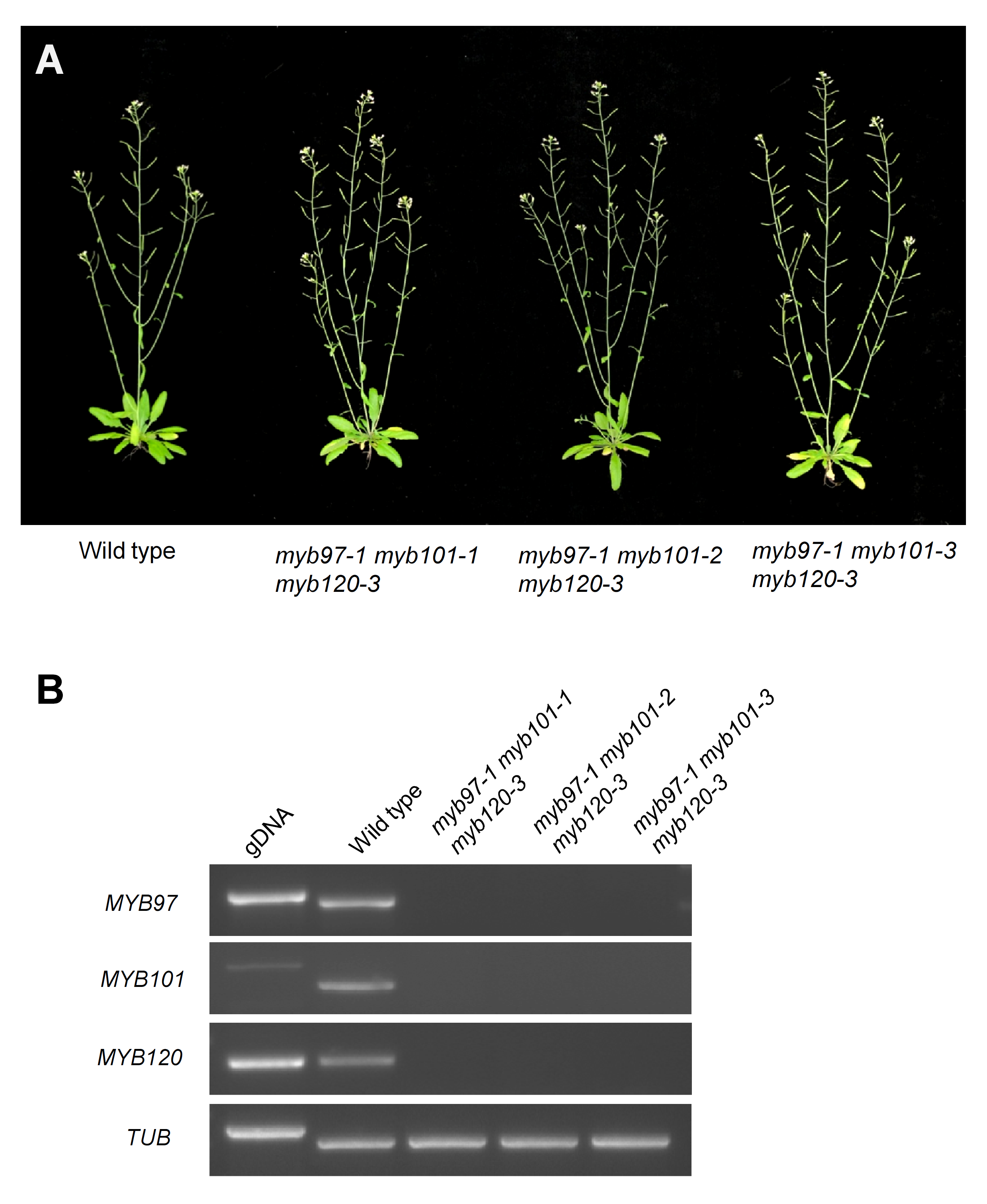

Supplement: Figure S2 — Isolation and characterization of the myb97 myb101 myb120 triple mutants. (A) The vegetative growth of the myb97 myb101 myb120 triple mutants is normal. (B) The transcript of MYB97, MYB101 and MYB120 in wild type and triple mutants, revealed by RT-PCR. (TIF) [file pgen.1003933.s002.tif]

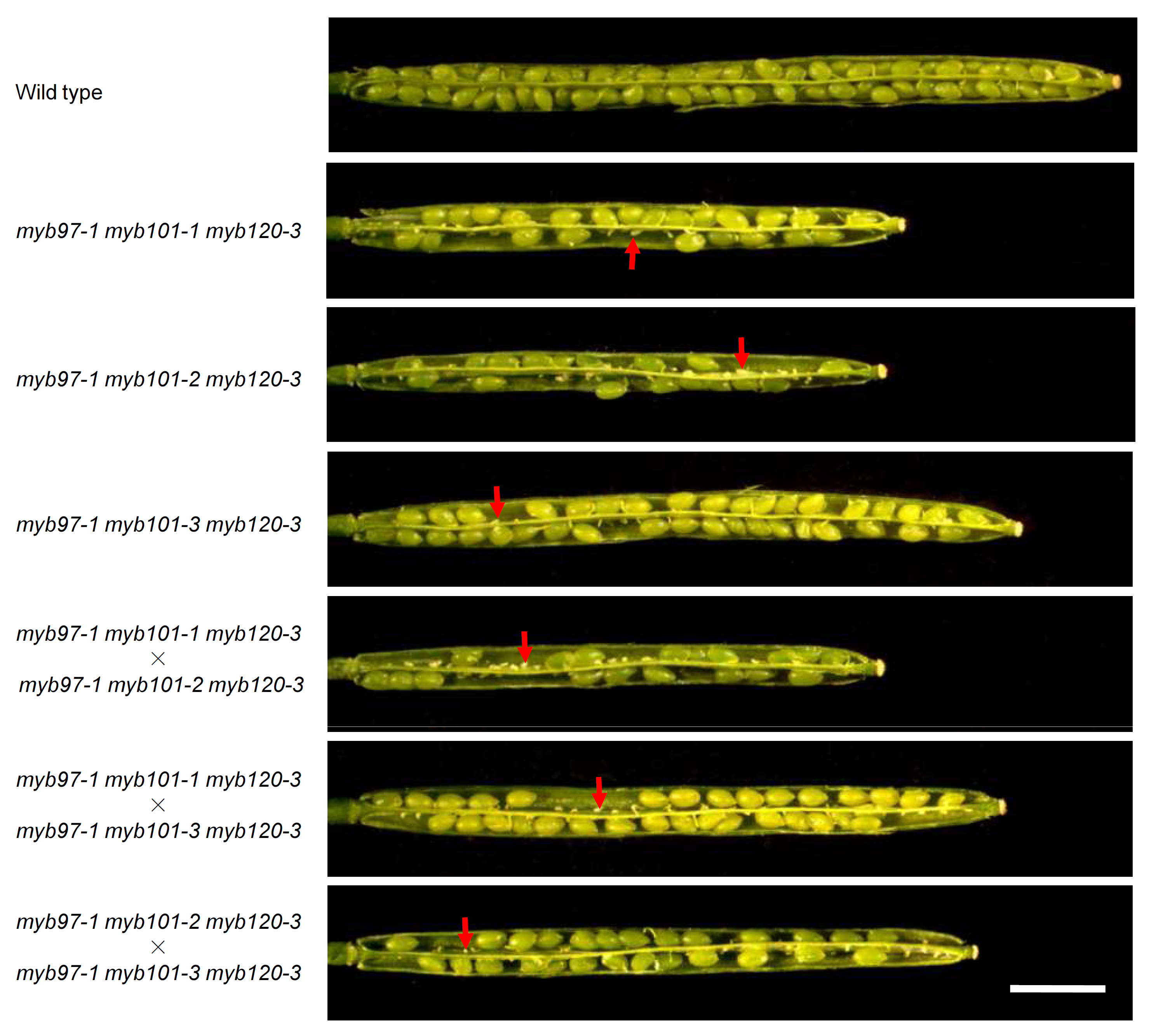

Supplement: Figure S3 — Phenotypic characterization and genetic analysis of the myb97 myb101 myb120 triple mutants. The triple myb97 myb101 myb120 mutants produced shorter siliques with reduced seed set compared to that of wild type. The red arrows indicate the unfertilized ovules. Bars = 2 mm. (TIF) [file pgen.1003933.s003.tif]

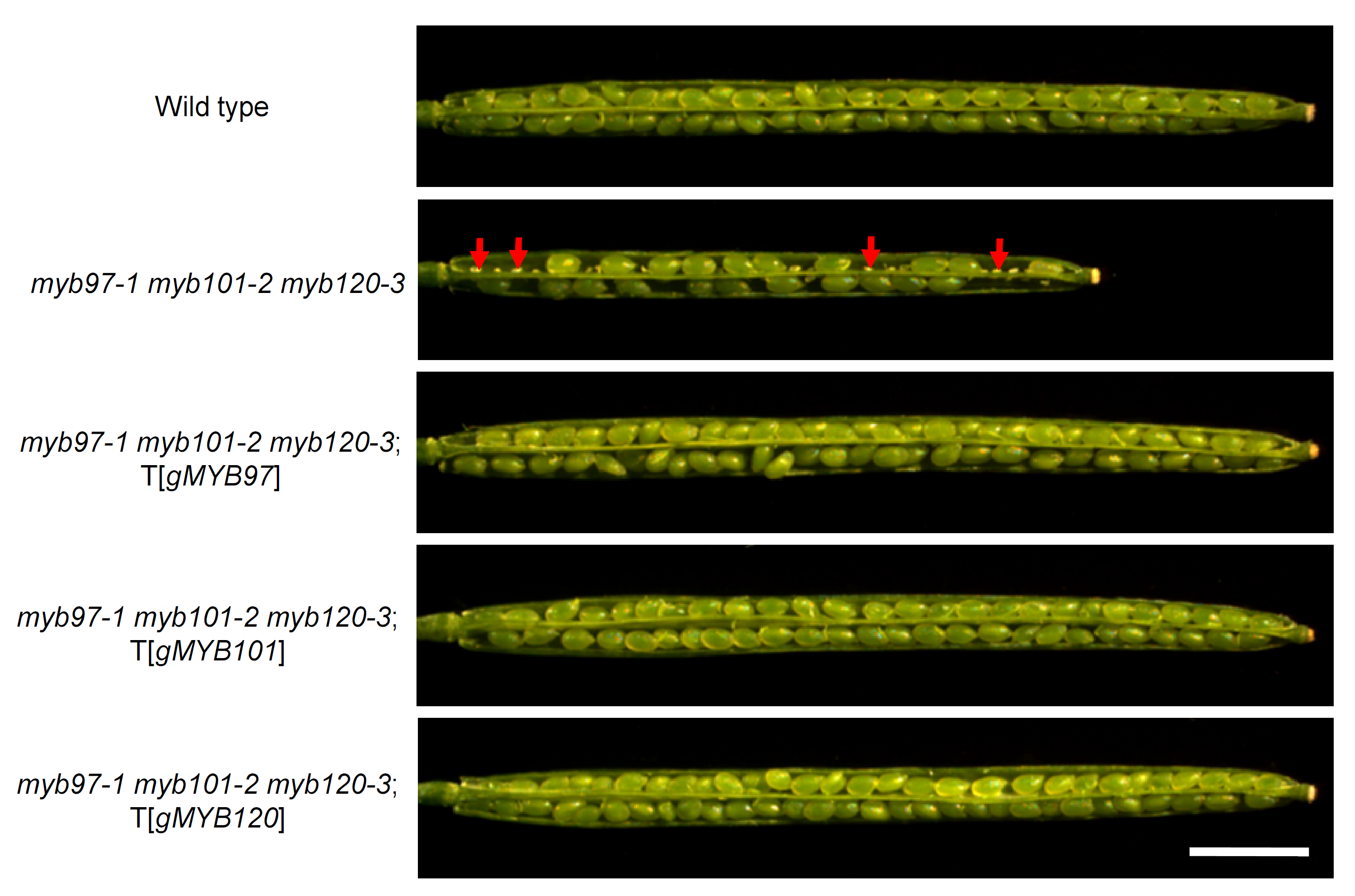

Supplement: Figure S4 — Complementation analysis of myb97-1 myb101-2 myb120-3 triple mutant by MYB97, MYB101 and MYB120 genomic DNAs. Transformation of MYB97, MYB101 and MYB120 complementation constructs could restore the fertility of the myb97-1 myb101-2 myb120-3 triple mutants completely. The red arrows indicate the unfertilized ovules. Bars =2 mm. (TIF) [file pgen.1003933.s004.tif]

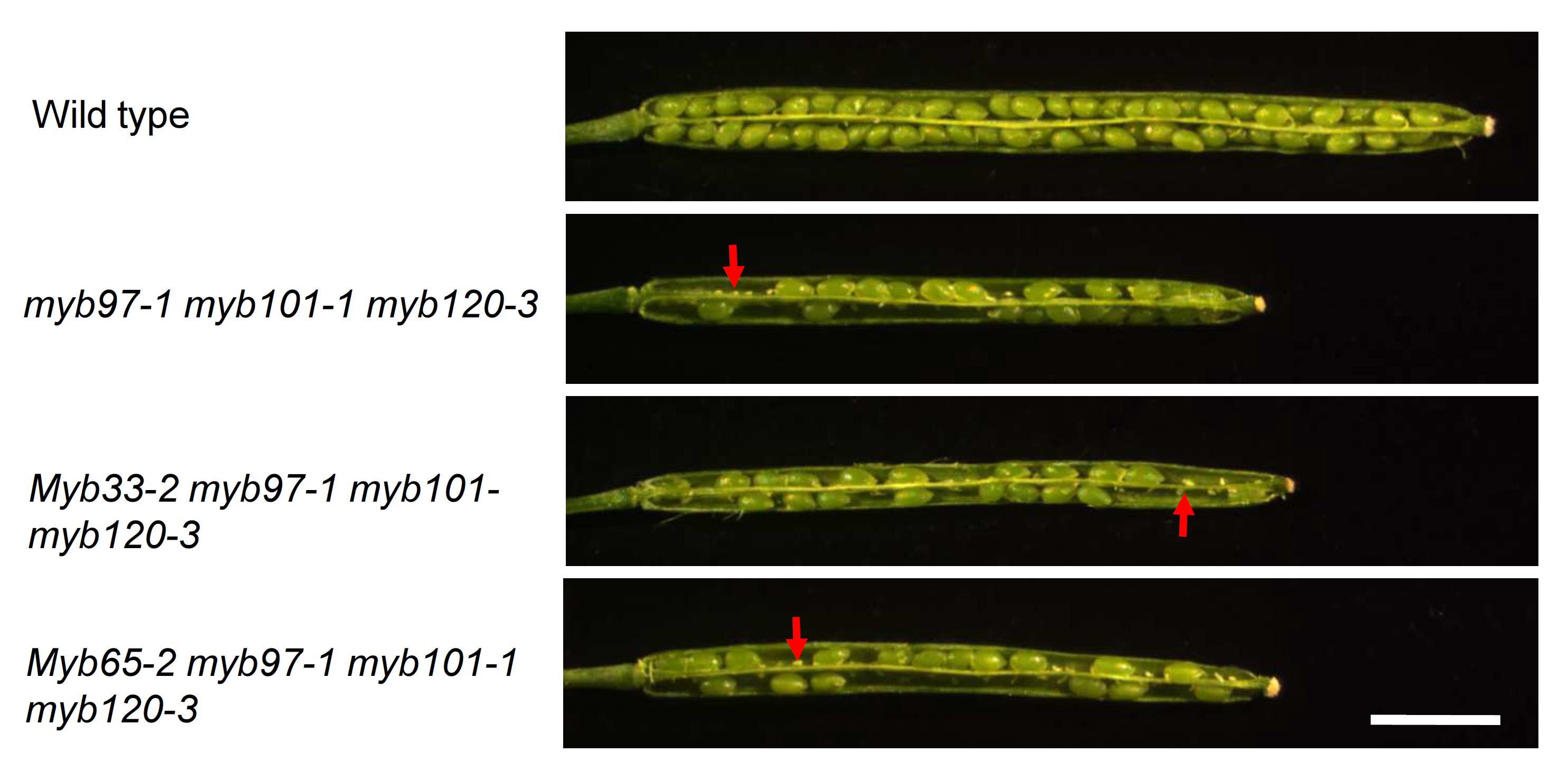

Supplement: Figure S5 — Phenotypic characterization of the quadruple myb mutants. The quadruple myb mutants did not exhibit more severe phenotypes compared to that of myb97-1 myb101-1 myb120-3 triple mutant. The red arrows indicate the unfertilized ovules. Bars = 2 mm. (TIF) [file pgen.1003933.s005.tif]
