# Supplementary material for: MYB97, MYB101 and MYB120 Function as Male Factors That Control Pollen Tube-Synergid Interaction in Arabidopsis thaliana Fertilization
Source: PLoS Genet. 2013 Nov 21;9(11):e1003933. doi: 10.1371/journal.pgen.1003933 (PMC3836714; doi:10.1371/journal.pgen.1003933)
Supplement: Table S1 — The MYB transcription factors involved in male gametophyte development in Arabidopsis. (DOCX) [file pgen.1003933.s006.docx]

**Table S1.** The MYB transcription factors involved in male gametophyte development in Arabidopsis

| Gene | Family | Function | Supplemental References (Text S1) |
| --- | --- | --- | --- |
| *TDF1* | R2R3-MYB | Tapetal differentiation and function | [1] |
| *MYB103* | R2R3-MYB | Tapetal development and microsporogenesis | [2, 3] |
| *MYB33/65* | R2R3-MYB | Tapetal development | [4] |
| *MYB4* | R2R3-MYB | Composition of pollen wall | [5] |
| *MYB32* | R2R3-MYB | Composition of pollen wall | [5] |
| *DUO1* | R2R3-MYB | Pollen mitosis | [6,7] |
| *MYB26* | R2R3-MYB | Anther dehiscence | [8] |
| *MYB21* | R2R3-MYB | Stamen filament growth | [9-11] |
| *MYB24* | R2R3-MYB | Stamen filament growth | [9-12] |
| *MYB57* | R2R3-MYB | Stamen filament growth | [10] |
