# Supplementary material for: MYB97, MYB101 and MYB120 Function as Male Factors That Control Pollen Tube-Synergid Interaction in Arabidopsis thaliana Fertilization
Source: PLoS Genet. 2013 Nov 21;9(11):e1003933. doi: 10.1371/journal.pgen.1003933 (PMC3836714; doi:10.1371/journal.pgen.1003933)
Supplement: Table S2 — Identification of the MYBs. (DOCX) [file pgen.1003933.s007.docx]

**Table S2.** Identification of the MYBs.

| MYB codes | GI |
| --- | --- |
| MYB33 | AT5G06100 |
| MYB65 | AT3G11440 |
| MYB81 | AT2G26960 |
| MYB97 | AT4G26930 |
| MYB101 | AT2G32460 |
| MYB104 | AT2G26950 |
| MYB120 | AT5G55020 |
