# Supplementary material for: MYB97, MYB101 and MYB120 Function as Male Factors That Control Pollen Tube-Synergid Interaction in Arabidopsis thaliana Fertilization
Source: PLoS Genet. 2013 Nov 21;9(11):e1003933. doi: 10.1371/journal.pgen.1003933 (PMC3836714; doi:10.1371/journal.pgen.1003933)
Supplement: Table S3 — The myb97 myb101 myb120 triple mutations reduced fertility. The statistics of silique length and seed set was performed in plants examined 50 days after transplantation into the soil; 30 siliques were examined for each combination. (DOCX) [file pgen.1003933.s008.docx]

**Table S3***.* The *myb97 myb101 myb120* triple mutations reduced fertility.

| Plants | | Silique Length  (cm) | Seed set (%) |
| --- | --- | --- | --- |
| Wild type | Col | 1.52 ± 0.03 | 99.42 ± 0.84 |
| Single mutants | *myb97-1* | 1.53 ± 0.04 | 98.81 ± 2.09 |
|  | *myb120-3* | 1.55 ± 0.07 | 98.67 ± 1.55 |
|  | *myb101-1* | 1.55 ± 0.05 | 99.74 ± 0.73 |
|  | *myb101-2* | 1.52 ± 0.03 | 98.71 ± 1.87 |
|  | *myb101-3* | 1.52 ± 0.07 | 99.73 ± 0.76 |
| Double mutants | *myb97-1 myb120-3* | 1.52 ± 0.04 | 99.43 ± 1.02 |
|  | *myb97-1 myb101-1* | 1.52 ± 0.04 | 99.77 ± 0.75 |
|  | *myb97-1 myb101-2* | 1.52 ± 0.04 | 99.77 ± 0.59 |
|  | *myb97-1 myb101-3* | 1.51 ± 0.03 | 99.72 ± 0.63 |
|  | *myb101-1 myb120-3* | 1.52 ± 0.05 | 99.48 ± 1.67 |
|  | *myb101-2 myb120-3* | 1.52 ± 0.08 | 99.60 ± 0.86 |
|  | *myb101-3 myb120-3* | 1.56 ± 0.05 | 99.72 ± 0.63 |
| Triple mutants | *myb97-1 myb101-1 myb120-3* | 1.14 ± 0.05 | 38.93 ± 6.13 |
|  | *myb97-1 myb101-2 myb120-3* | 1.06 ± 0.06 | 36.61 ± 6.13 |
|  | *myb97-1 myb101-3 myb120-3* | 1.33 ± 0.04 | 72.75 ± 6.13 |
| Crosses (Female X Male) | *myb97-1 myb101-1 myb120-3* X *myb97-1 myb101-2 myb120-3* | 1.09 ± 0.05 | 39.03 ± 5.38 |
|  | *myb97-1 myb101-1 myb120-3* X *myb97-1 myb101-3 myb120-3* | 1.16 ± 0.05 | 55.39 ± 6.31 |
|  | *myb97-1 myb101-2 myb120-3* X *myb97-1 myb101-3 myb120-3* | 1.21 ± 0.05 | 54.68 ± 6.46 |

The statistics of silique length and seed set was performed in plants examined 50 days after transplantation into the soil; 30 siliques were examined for each combination.
