# Supplementary material for: MYB97, MYB101 and MYB120 Function as Male Factors That Control Pollen Tube-Synergid Interaction in Arabidopsis thaliana Fertilization
Source: PLoS Genet. 2013 Nov 21;9(11):e1003933. doi: 10.1371/journal.pgen.1003933 (PMC3836714; doi:10.1371/journal.pgen.1003933)
Supplement: Table S4 — Complementation analysis of the myb97-1 myb101-2 myb120-3 homozygous mutant. The statistics of silique length and seed set was performed in plants examined 50 days after transplantation into the soil. a, 30 siliques were examined; b, 75 siliques from 5 independent transgenic plants were examined. T[gMYB97], transgenic MYB97; T[gMYB101], transgenic MYB101; T[gMYB120], transgenic MYB120. (DOCX) [file pgen.1003933.s009.docx]

**Table S4**. Complementation analysis of the *myb97-1 myb101-2* *myb120-3* homozygous mutant.

| Genotypes | Silique Length (cm) | Seed set (%) |
| --- | --- | --- |
| WT^a^ | 1.58 ± 0.05 | 98.86 ± 1.55 |
| *myb97-1 myb101-2 myb120-3*^a^ | 1.02 ± 0.06 | 31.97 ± 4.98 |
| *myb97-1 myb101-2 myb120-3;* T[*gMYB97*]^b^ | 1.50 ± 0.04 | 99.59 ± 1.07 |
| *myb97-1 myb101-2 myb120-3;* T[*gMYB101*] ^b^ | 1.42 ± 0.04 | 98.73 ± 1.82 |
| *myb97-1 myb101-2 myb120-3;* T[*gMYB120*] ^b^ | 1.45 ± 0.05 | 98.54 ± 2.29 |

The statistics of silique length and seed set was performed in plants examined 50 days after transplantation into the soil. a, 30 siliques were examined; b, 75 siliques from 5 independent transgenic plants were examined. T[*gMYB97*], transgenic *MYB97*; T[*gMYB101*], transgenic *MYB101*; T[*gMYB120*], transgenic *MYB120.*
