# Supplementary material for: MYB97, MYB101 and MYB120 Function as Male Factors That Control Pollen Tube-Synergid Interaction in Arabidopsis thaliana Fertilization
Source: PLoS Genet. 2013 Nov 21;9(11):e1003933. doi: 10.1371/journal.pgen.1003933 (PMC3836714; doi:10.1371/journal.pgen.1003933)
Supplement: Table S5 — Genetic analysis of the myb97-1 myb101-2 myb120-3 heterozygous mutant. W, with T-DNA; Wo, without T-DNA; TE, transmission efficiency: (W∶Wo)×100%; TEF, female transmission efficiency; TEM, male transmission efficiency; NA, not applicable; +/+, wild type; myb97-1/−, homozygous myb97-1; myb97-1/+, heterozygous myb97-1; The same format is used for myb101-1 and myb120-3. (DOCX) [file pgen.1003933.s010.docx]

**Table S5.** Genetic analysis of the *myb97-1 myb101-2 myb120-3* heterozygous mutant.

| Crosses (Female X Male) | W | Wo | W:Wo | TE_F_ (%) | TE_M_ (%) |
| --- | --- | --- | --- | --- | --- |
| *myb97-1/+*;*myb101-2/*-;*myb120-3/-* (self) | 98 | 67 | 1.46:1 | NA | NA |
| +/+ X *myb97-1/+*;*myb101-2/*-;*myb120-3/-* | 21 | 156 | 0.13:1 | NA | 13 |
| *myb97-1/+*;*myb101-2/*-;*myb120-3/-* X *+/+* | 97 | 90 | 1.08:1 | 100 | NA |
| *myb97-1/-*;*myb101-2/*-;*myb120-3/+* (self) | 77 | 65 | 1.18:1 | NA | NA |
| +/+ X *myb97-1/-*;*myb101-2/*-;*myb120-3/+* | 13 | 173 | 0.08:1 | NA | 8 |
| *myb97-1/-*;*myb101-2/*-;*myb120-3/+* X *+/+* | 82 | 79 | 1.04:1 | 100 | NA |
| *myb97-1/-;myb101-2/*+;*myb120-3/-* (self) | 231 | 193 | 1.20:1 | NA | NA |
| +/+ X *myb97-1/-;myb101-2/*+;*myb120-3/-* | 19 | 233 | 0.08:1 | NA | 8 |
| *myb97-1/-;myb101-2/*+;*myb120-3/-* X *+/+* | 138 | 146 | 0.95:1 | 95 | NA |

W, with T-DNA; Wo, without T-DNA; TE, transmission efficiency: (W:Wo) X 100%; TE_F_, female transmission efficiency; TE_M_, male transmission efficiency; NA, not applicable; +/+, wild type; *myb97-1/-*, homozygous *myb97-1*; *myb97-1/+,* heterozygous *myb97-1*; The same format is used for *myb101-1* and *myb120-3*.
