# Supplementary material for: MYB97, MYB101 and MYB120 Function as Male Factors That Control Pollen Tube-Synergid Interaction in Arabidopsis thaliana Fertilization
Source: PLoS Genet. 2013 Nov 21;9(11):e1003933. doi: 10.1371/journal.pgen.1003933 (PMC3836714; doi:10.1371/journal.pgen.1003933)
Supplement: Table S6 — The genes whose expression was affected in the myb97-1 myb101-1 myb120-3 triple mutant, revealed by microarray analysis. Fold change = Log2(mutant/WT). (DOCX) [file pgen.1003933.s011.docx]

**Table S6.** The genes whose expression was affected in the *myb97-1 myb101-1 myb120-3* triple mutant, revealed by microarray analysis.

| AGI | Description | Fold change |
| --- | --- | --- |
| Downregulated Genes (DG) | | |
| *AT5G66300* (*DG1*) | NAC105 (NAC-domain transcription factor) | -5.69 |
| *AT1G69840* (*DG2*) | SPFH/Band 7/PHB domain-containing membrane-associated protein | -3.42 |
| *AT3G19690* (*DG3*) | Secretory protein | -4.34 |
| *AT3G12580* (*DG4*) | HSP70 (heat shock protein) | -1.18 |
| *AT2G28590* | Putative protein kinase | -1.09 |
| *AT3G07490* | AGD11 (ARF-GAP DOMAIN 11) | -1.24 |
| *AT5G24655* | LSU4 ( RESPONSE TO LOW SULFUR 4) | -1.2 |
| *AT4G36680* | TPR (Tetratricopeptide repeat)-like protein | -1.32 |
| Upregulated Genes (UG) | | |
| *AT2G06850* | Endoxyloglucan transferase | 2.17 |
| *AT2G24210* | TPS10 (TERPENE SYNTHASE 10) | 1.62 |
| *AT1G07400 /// AT1G59860* | HSP20-like chaperones superfamily protein | 1.53 |
| *AT1G14200* | RING/U-box protein | 1.18 |
| *AT1G16030* | HSP70B (HEAT SHOCK PROTEIN 70B) | 1.11 |
| *AT1G62380* | ACO2 (ACC OXIDASE 2) | 2.04 |
| *AT1G74450* | Unknown protein | 2.71 |
| *AT3G28210* | Putative zinc finger protein | 1.42 |
| *AT4G30270* | Protein similar to endo xyloglucan transferase | 2.15 |
| *AT3G48360* | BT2 ( BTB and TAZ domain protein 2) | 1.85 |
| *AT3G53300* | Putative cytochrome P450 | 3.33 |
| *AT5G05410* | DREB2A transcription factor | 1.27 |
| *AT5G44060* | Unknown protein | 1.23 |
| *AT5G45950* | GDSL-like Lipase/Acylhydrolase protein | 3.14 |
| *AT5G48570* | CC (Carboxylate clamp)- TPR (tetratricopeptide repeat) protein | 1.14 |
| *AT1G51060* | Histone H2A protein | 1.17 |

Fold change = Log_2_(mutant/WT).
