# Supplementary material for: MYB97, MYB101 and MYB120 Function as Male Factors That Control Pollen Tube-Synergid Interaction in Arabidopsis thaliana Fertilization
Source: PLoS Genet. 2013 Nov 21;9(11):e1003933. doi: 10.1371/journal.pgen.1003933 (PMC3836714; doi:10.1371/journal.pgen.1003933)
Supplement: Table S7 — Phenotypic analysis of the quadruple myb mutants. The statistics of silique length and seed set was performed in plants examined 50 days after transplantation into the soil; 30 siliques were examined for each combination. (DOCX) [file pgen.1003933.s012.docx]

**Table S7***.* Phenotypic analysis of the quadruple *myb* mutants.

| Genotypes | Silique Length (cm) | Seed set (%) |
| --- | --- | --- |
| WT | 1.58 ± 0.07 | 97.30 ± 3.78 |
| *myb97-1 myb101-1 myb120-3* | 1.13 ± 0.07 | 29.50 ± 4.36 |
| *myb33-2 myb97-1 myb101-1 myb120-3* | 1.10 ± 0.07 | 29.81 ± 5.50 |
| *myb65-2 myb97-1 myb101-1 myb120-3* | 1.10 ± 0.06 | 31.71 ± 5.58 |

The statistics of silique length and seed set was performed in plants examined 50 days after transplantation into the soil ; 30 siliques were examined for each combination.
