# Supplementary material for: MYB97, MYB101 and MYB120 Function as Male Factors That Control Pollen Tube-Synergid Interaction in Arabidopsis thaliana Fertilization
Source: PLoS Genet. 2013 Nov 21;9(11):e1003933. doi: 10.1371/journal.pgen.1003933 (PMC3836714; doi:10.1371/journal.pgen.1003933)
Supplement: Table S8 — Sequences of the primers used in this study. (DOCX) [file pgen.1003933.s013.docx]

**Table S8**. Sequences of the primers used in this study.

| Primers | Sequences (5’-3’) | Enzyme |
| --- | --- | --- |
| LBa1 | TGGTTCACGTAGTGGGCCATCG |  |
| MYB97-1-RP | TAGTTACCAGGCAGAACAGATAACG |  |
| MYB97-1-LP | TGGGATGTCCACTAGCATGTAAC |  |
| MYB120-3-RP | TCAAATCTGCGTTTTGATC |  |
| MYB120-3-LP | CGAAACACACCATCATCTTC |  |
| MYB101-1-RP | CGCTATTGTCTAGTCCTAAAGG |  |
| MYB101-1-LP | CTTCAATTCAAGAAACTAGAGGGC |  |
| MYB101-2-RP | GATTTAACTTCCTTTCAGTTCC |  |
| MYB101-2-LP | CGATACCAATCAGGCAAAGGT |  |
| MYB101-3-RP | CTGTGTAAGAGGATCTGATAAATC |  |
| MYB101-3-LP | CCTTCTGGCAATAGTGGACTACTT |  |
| TUB-RT-F | GGACACTACACTGAAGGTGCTGAG |  |
| TUB-RT-R | GGCTCTGTATTGCTGTGATCCACG |  |
| MYB97-RT-F | TGGAATTCTGTTCAGAAGAAG |  |
| MYB97-RT-R | GGATCCCAAGTTGTTGGTGTCATCATC |  |
| MYB120-RT-F | GTTTCAAGAGACTCCTACAC |  |
| MYB120-RT-R | TCTAGAAGCACCGGAACTGTTAAG |  |
| MYB101-RT-F | CACGCTAAGCTTGGAAAC |  |
| MYB101-RT-R | GGATCCACAGATGCTAGGCATGTTGCT |  |
| MYB33-RT-F | TCGTCATCTCCTCCACACTCTG |  |
| MYB33-RT-R | TCGCTCATGCTACCTGTCTCT |  |
| MYB65-RT-F | TGTTACTCAGAATGTACCACGTGGCAG |  |
| MYB65-RT-R | TACAGCGACCAAACAGGAGGCA |  |
| MYB81-RT-F | CTTCATGCTTTGTTAGGGAAC |  |
| MYB81-RT-R | AAGGATAGTTCCAGAGCCGA |  |
| MYB104-RT-F | TCCTCATAATCCTGCAAGTTGTCG |  |
| MYB104-RT-R | TGGTTCCATAACGCGATTGCG |  |
| ACTIN2-rl-F | GGTAACATTGTGCTCAGTGGTGG |  |
| ACTIN2-rl-R | AACGACCTTAATCTTCATGCTGC |  |
| MYB97-rl-F | ATCATACAACTCCACTCTCAGC |  |
| MYB97-rl-R | CTCGTTATCTGTTCTGCCTGG |  |
| MYB101-rl-F | GGCGGACTCTTCAAGGAC |  |
| MYB101-rl-R | GTTGTGAATATTAGGGTTTGCTCC |  |
| MYB120-rl-F | GTAACAAATGGGCTCGCATG |  |
| MYB120-rl-R | GGATGGAGTTGATGGTTAGGG |  |
| MYB33-rl-F | TGTATCCCAAACTAAATCCGAGG |  |
| MYB33-rl-R | CGTCAGGCCTTGTTATATCCAAG |  |
| MYB65-rl-F | AGATTACAAGTCCCTCGCAAC |  |
| MYB65-rl-R | CAAGTAAAACATCAGGCCGTG |  |
| MYB81-rl-F | TCGCCTTCGTTGGGTAAATC |  |
| MYB81-rl-R | CATTATCTGTTCGTCCCGGTAG |  |
| MYB104-rl-F | CGATGAGGAAGAGAAGCGTG |  |
| MYB104-rl-R | ACCCTTTCCTTTCAATCGCA |  |
| DG1-rl-F | ACGCTTGTCTTTTACCGAGG |  |
| DG1-rl-R | CACACTACCCATCCTTCTTCC |  |
| DG2-rl-F | TGATTGTGGATATCGAGCCTG |  |
| DG2-rl-R | TTTCGATTCAGCTTCTCCCTC |  |
| DG3-rl-F | ATGCTGCGGAAATGTGGATC |  |
| DG3-rl-R | GCACATCCCAAACGAACTGT |  |
| DG4-rl-F | GTGCTTGACTATCCCTTCTGG |  |
| DG4-rl-R | GTCTGAATCTCCACCTGATCG |  |
| MYB97-GUS-F | CACTGCAGCGATATTGTCACCGTATAGC | *Pst* I |
| MYB97-GUD-R | GGATCCGGTGATTGGTAGAACGTC | *Bam*H I |
| MYB120-GUS-F | ATCTGCAGCTGATGCTCCACCGAAG | *Pst* I |
| MYB120-GUS-R | TCTAGAAGCTTCTTCCCTTGCCGA | *Xba* I |
| MYB101-GUS-F | CTGCAGACACCAAATACATGTGGT | *Pst* I |
| MYB101-GUS-R | GGATCCTCCTTTTCAACACGGCGA | *Bam*H I |
| MYB97-F-1F | CACTGCAGCGATATTGTCACCGTATAGC | *Pst* I |
| MYB97-F-1R | CAGAATTCCAGTTCCCTTCAC | *Eco*R I |
| MYB97-F-2F | TGGAATTCTGTTCAGAAGAAG | *Eco*R I |
| MYB97-F-2R | ATGGTACCATGTTCATCGTAGGTGTGG | *Kpn* I |
| MYB120-F-1F | ATCTGCAGCTGATGCTCCACCGAAG | *Pst* I |
| MYB120-F-1R | CGCCGTCCATGGACCTTTCTT | *Nco* I |
| MYB120-F-2F | AAGAAAGGTCCATGGACGGCG | *Nco* I |
| MYB120-F-2R | TAGAGCTCATGACTGCGTCTAGGTAG | *Sac* I |
| MYB101-F-1F | CTGCAGACACCAAATACATGTGGT | *Pst* I |
| MYB101-F-1R | GTTTCCAAGCTTAGCGTG | *Hin*d III |
| MYB101-F-2F | CACGCTAAGCTTGGAAAC | *Hin*d III |
| MYB101-F-2R | GAGCTCAGATCCATATGTACCATG | *Sac* I |
| MYB33-T-F | GCGGATCCATGAGTTACACGAGCACTGACAGT | *Bam*H I |
| MYB33-T-R | TCCCGGGTTAGGGTAGTTCTGTCATTTGACAG | *Sma* I |
| MYB81-T-F | GCGGATCCACAGATGACACAAGATGG | *Bam*H I |
| MYB81-T-R | GAGAGCTCTCAAAGGATGTGTGTCCCTGA | *Sac* I |
| MYB97-G-F | GCTCTAGAATGATCGTGTACGGTGGG | *Xba* I |
| MYB97-G-R | TAGGTACCCTAGCAGATCCCTGGCAAGTTG | *Kpn* I |
| MYB120-G-F | GTCTAGAATGATCATGTACGGAGGAGGAGGAGCAG | *Xba* I |
| MYB120-G-R | CGGTCGACGTCAAATCTGCGTTTTGATC | *Sal* I |
| MYB101-G-F | GCTCTAGAATGGATGGTGGTGGAGAGACGAC | *Xba* I |
| MYB101-G-R | GAGAGCTCCTAACAGATGCTAGGCATG | *Sac* I |
| AD-F | AAAGGATCCAAGCGGAATTAATTCCCGAG | *Bam*H I |
| AD-R | AAAGTCGACCCTCTTTTTTTGGGTTTGGTGG | *Sal* I |
| MYB97-BD-F | GCCATATGATGATCGTGTACGGTGGG | *Nde* I |
| MYB120-BD-F | GGAATTCATGATCATGTACGGAGGAGGAGGAGCAG | *Eco*R I |
| MYB101-BD-F | GCCATATGATGGATGGTGGTGGAGAGACGAC | *Nde* I |
| MYB101-P-F | CGCGGATCCATGGATGGTGGTGGAGAG | *Bam*H I |
| MYB101-P-R | ACGCGTCGACAATCTCATGAGGGTATAA | *Sal* I |
