# Supplementary material for: MYB97, MYB101 and MYB120 Function as Male Factors That Control Pollen Tube-Synergid Interaction in Arabidopsis thaliana Fertilization
Source: PLoS Genet. 2013 Nov 21;9(11):e1003933. doi: 10.1371/journal.pgen.1003933 (PMC3836714; doi:10.1371/journal.pgen.1003933)
Supplement: Text S1 — Supplemental references. (DOCX) [file pgen.1003933.s014.docx]

**Text S1**

**Supplemental References**

1. Zhu J, Chen H, Li H, Gao JF, Jiang H, et al. (2008) Defective in tapetal development and function 1 is essential for anther development and tapetal function for microspore maturation in *Arabidopsis*. Plant J 55: 266-277.

2. Higginson T, Li SF, Parish RW (2003) AtMYB103 regulates tapetum and trichome development in *Arabidopsis thaliana*. Plant J 35: 177-192.

3. Zhang ZB, Zhu J, Gao JF, Wang C, Li H, et al. (2007) Transcription factor AtMYB103 is required for anther development by regulating tapetum development, callose dissolution and exine formation in *Arabidopsis*. Plant J 52: 528-538.

4. Millar AA, Gubler F (2005) The *Arabidopsis* GAMYB-like genes, MYB33 and MYB65, are microRNA-regulated genes that redundantly facilitate anther development. Plant Cell 17: 705-721.

5. Preston J, Wheeler J, Heazlewood J, Li SF, Parish RW (2004) AtMYB32 is required for normal pollen development in *Arabidopsis thaliana*. Plant J 40: 979-995.

6. Rotman N, Durbarry A, Wardle A, Yang WC, Chaboud A, et al. (2005) A novel class of MYB factors controls sperm-cell formation in plants. Curr Biol 15: 244-248.

7. Brownfield L, Hafidh S, Borg M, Sidorova A, Mori T, et al. (2009) A plant germline-specific integrator of sperm specification and cell cycle progression. PLoS Genet 5: e1000430.

8. Steiner-Lange S, Unte US, Eckstein L, Yang C, Wilson ZA, et al. (2003) Disruption of A*rabidopsis thaliana* *MYB26* results in male sterility due to non-dehiscent anthers. Plant J 34: 519-528.

9. Mandaokar A, Thines B, Shin B, Lange BM, Choi G, et al. (2006) Transcriptional regulators of stamen development in *Arabidopsis* identified by transcriptional profiling. Plant J 46: 984-1008.

10. Cheng H, Song S, Xiao L, Soo HM, Cheng Z, et al. (2009) Gibberellin acts through jasmonate to control the expression of MYB21, MYB24, and MYB57 to promote stamen filament growth in *Arabidopsis*. PLoS Genet 5: e1000440.

11. Song S, Qi T, Huang H, Ren Q, Wu D, et al. (2011) The Jasmonate-ZIM domain proteins interact with the R2R3-MYB transcription factors MYB21 and MYB24 to affect jasmonate-regulated stamen development in Arabidopsis. Plant Cell 23: 1000-1013.

12. Yang XY, Li JG, Pei M, Gu H, Chen ZL, et al. (2007) Over-expression of a flower-specific transcription factor gene *AtMYB24* causes aberrant anther development. Plant Cell Rep 26: 219-228.
